# Using multiple Mendelian randomization approaches and genetic correlations to understand obesity, urate, and gout

**DOI:** 10.1038/s41598-021-97410-4

**Published:** 2021-09-07

**Authors:** Charleen D. Adams, Brian B. Boutwell

**Affiliations:** 1 Department of Environmental Health, Program in Molecular and Integrative Physiological Sciences, Harvard T.H. Chan School of Public Health, Boston, MA 02115 USA; 2 School of Applied Science, The University of Mississippi, P.O. Box 1848, University, MS 38677 USA; 3 John D. Bower School of Population Health, University of Mississippi Medical Center, Jackson, MS 39216 USA

**Keywords:** Nutrition disorders, Obesity, Genetics

## Abstract

Observational studies suggest relationships between obesity, urate, and gout but are possibly confounded. We assessed whether genetically determined obesity, higher urate (and related traits), and gout were causal using multiple Mendelian randomization (MR) approaches and linkage disequilibrium score regression for genetic correlations (*r*_*g*_). For data, we used genome-wide association study summary statistics available through MR-Base. We observed that obesity increased urate (beta = 0.127; 95% CI = 0.098, 0.157; *P*-value = 1.2E−17; *r*_*g*_ = 0.25 [*P*-value = 0.001]) and triglycerides (beta = 0.082; 95% CI = 0.065, 0.099; *P*-value = 1.2E−21; *r*_*g*_ = 0.23 [*P*-value = 8.8E−12]) and decreased high-density lipoprotein cholesterol (HDL) (beta = − 0.083; 95% CI = − 0.101, − 0.065; *P*-value = 2.5E−19; *r*_*g*_ = − 0.28; [*P*-value = 5.2E−24]). Higher triglycerides increased urate (beta = 0.198; 95% CI = 0.146, 0.251; *P*-value = 8.9E−14; *r*_*g*_ = 0.29 [*P*-value = 0.001]) and higher HDL decreased urate (beta = − 0.109; 95% CI = − 0.148, − 0.071; *P*-value = 2.7E− 08; *r*_*g*_ = − 0.21 [*P*-value = 9.8E−05]). Higher urate (OR = 1.030; 95% CI = 1.028, 1.032; *P*-value = 1.1E−130; *r*_*g*_ = 0.89 [*P*-value = 1.7E−55]) and obesity caused gout (OR = 1.003; 95% CI = 1.001, 1.004; *P*-value = 1.3E−04; *r*_*g*_ = 0.23 [*P*-value = 2.7E−05]). Obesity on gout with urate as a mediator revealed all the effect of obesity on gout occurred through urate. Obesity on low-density lipoprotein cholesterol (LDL) was null (beta = −0.011; 95% CI = −0.030, 0.008; *P*-value = 2.6E−01; *r*_*g*_ = 0.03 [*P*-value = 0.369]). A multivariable MR of obesity, HDL, and triglycerides on urate showed obesity influenced urate when accounting for HDL and triglycerides. Obesity’s impact on urate was exacerbated by it decreasing HDL.

## Introduction

Human diets, spanning from ancestral to current, involve substantive consumption of foods which can increase a waste product of human metabolism known as urate^1^. This is owed largely to the prevalence of meat and fish (which contain purines), but also to the consumption of fructose and glucose-rich foods^1^. The possible downstream consequences of elevated urate have interested researchers across fields for some time^1,2^. The most widely examined consequence of urate production involves gout, an inflammatory arthritic disease reflecting the accumulation of crystallized urate in joints, most prominently in the largest toe^3^. Correlations exist too, however, between serum urate and cardiovascular disease, chronic kidney disease, and metabolic syndrome, an outcome representing a confluence of connected risk factors (e.g., obesity, type 2 diabetes [T2D], etc.)^2^. For patients with coronary artery disease in particular some associational evidence suggests that urate may represent an independent risk factor for mortality^4^.

Moreover, genetic correlations between serum urate and body mass index (BMI) are modest but positive and range from 0.20 to 0.28^5^, and a robust canon of epidemiologic literature suggests consistent relationships between obesity, urate, and gout^6–10^. Using the National Health and Nutrition Surveys (NHANES) data on adult participants in the US from 1988–1994 and 2007–2010, for instance, researchers estimated the prevalence of gout among participants of normal BMI to be 1–2%, but 4–5% in those classified with class I obesity and 5–7% in those with class II or III obesity^11^. In a population-based study of men in Japan, ﻿﻿visceral fat accumulation was identified in 56% of men with hyperuricemia, hinting at the role of obesity in potentially causing higher urate levels^8^.

Complicating research on this topic is the common inability to disentangle cause from effect^3^. Epidemiological designs can provide insight about risk, yet accounting for potential reverse causation, as well as non-random selection across environmental, lifestyle, dietary, and genetic risk factors produce a range of challenges, none easily surmounted^12^. Mendelian randomization (MR), however, which utilizes genome-wide associational (GWA) studies to build genetic “instruments” for putative environmental risk factors, like urate, offers a way to overcome many of these difficulties.

In a recent study of T2D, for example, Sluijs and colleagues (2015) failed to find a causal effect of urate levels on T2D when utilizing MR designs, despite replicating the previously apparent epidemiological correlations^3^. The analysis by Sluijs et al. (2015) is an important step in better understanding one of the possible health risks imposed by elevated urate levels. Still, several interesting hypotheses remain. Elevated urate, for example, might be “an effect” of other metabolic disorders—such as obesity—rather than a straightforward causal agent. Here we combine several MR designs to zoom in on the roles of genetically determined obesity, urate, and gout. We conceive of the MR analysis as having four parts:

1. Univariable MR of genetically determined obesity on four traits: circulating levels of high-density lipoprotein cholesterol (HDL), low-density lipoprotein cholesterol (LDL), triglycerides, and urate.
2. Univariable MR of three trait-levels (genetically determined HDL, triglycerides, and urate) on gout.
3. Multivariable MR analysis of genetically determined obesity, HDL, and triglycerides in relation to urate levels.
4. Mediation MR analysis of the total, direct, and indirect effects of genetically determined obesity on gout conceiving of urate as the mediator.

In addition to the MR analysis, we performed linkage disequilibrium (LD) score regression for genetic correlations (*r*_*g*_) for pairs of traits in the univariable models. Genetic correlations between obesity and five traits were calculated: obesity and HDL, obesity and LDL, obesity and triglycerides, obesity and urate, and obesity and gout. Genetic correlations between urate and four traits (urate and HDL, urate and LDL, urate and triglycerides, and urate and gout) were also calculated. The purpose for complementing the MR analysis with genetic correlations is that genetic correlations provide a measure of pleiotropy (defined as a genetic locus influencing more than one trait)^13^, and (vertical) pleiotropy is a necessary condition for causality between two genetically determined traits. The pleiotropy captured by genetic correlations can be due to different modes of action. It can be, as just-mentioned, vertical (also called “mediated”), the type of pleiotropy MR exploits. It can also be horizontal (considered “unwanted”), the type of pleiotropy that causes a violation to MR assumptions (discussed in Methods). With horizontal pleiotropy, a genetic variant contributes to multiple traits directly or indirectly to them through a third (intermediate) trait. Finally, pleiotropy can be spurious (due to a genetic variant being in LD with other genetic variants that are causal for the traits). Thus, a measure of genetic correlation between two traits permits an appraisal of the existence of pleiotropy^13^, which, if vertical, provides evidence that the traits may have an underlying causal relationship.

## Methods

### Conceptual approach

MR is an instrumental variables technique. It uses insights from Mendel’s laws of inheritance and genotype assignment at conception^14–16^ to improve causal inference in epidemiologic studies. It does this by using genetic variants reliably associated with traits of interest in statistical models instead of the traits themselves (i.e., genetic variants, typically single-nucleotide polymorphisms, SNPs, are used as “instruments”). Doing so prevents much of the confounding and reverse causation that bias estimates in observational studies, so long as certain assumptions are not violated.

### MR assumptions

Key MR assumptions include the following (see^14–16^):

1. The SNPs serving as genetic instruments must be reliably associated with the “exposure” (independent variable) of interest.
2. The instrumental SNPs must not be associated with confounders of the exposure-“outcome” (dependent variable) relationship.
3. There must not be any other pathway from the SNP to the outcome other than through the exposure (i.e., no horizontal pleiotropy).

In two-sample MR, summary statistics (e.g., effect estimates, standard errors, and *P*-values) are the data sources^17–22^. Readers less familiar with techniques in genetic epidemiology may confuse individual genotype calls with summary data. To ensure there is not confusion, the effect estimates referred to as “summary statistics” are not the genotypes for individuals but the effects for the associations between SNPs and phenotypes in a population—they are the results of GWA studies. Using a two-sample MR design to calculate the causal effect of obesity on gout, for example, estimates of SNP-obesity associations (*β*^ZX) are calculated in sample 1 (from a GWA study of obesity). The association between these same SNPs and gout is then estimated in sample 2 (*β*^ZY) (from a GWA study of gout). These estimates are combined into Wald ratios (*β*^XY = *β*^ZY/β^ZX). The *β*^XY estimates are meta-analyzed using the inverse-variance weighted (IVW) method and various sensitivity estimators. The IVW method produces an overall causal estimate for obesity on gout.

In the present study, summary statistics were downloaded from MR-Base (http://www.mrbase.org/)^17^, a repository of freely available GWA study results (see Supplementary tables 10–18). Descriptions of the GWA studies and the MR analyses they were used for are in Table 1. (A note to readers: In Table 1 and in what follows, continuous traits (i.e., levels of urate, HDL, LDL, and triglycerides) are sometimes referred to without the word “levels” for ease of reading).

**Table 1:** Univariable MR analyses and GWA data sources.

MR analysis	Instrument data source	Outcome data source
Obesity on urate	Berndt et al*.* (2013)^23^: a meta-analysis of GWA studies of clinically defined obesity in 98,679 participants of European ancestry, of which 32,858 were classified as obese. Obesity was defined as BMI ≥ 30 kg/m^2^. Analyses accounted for sex and used a genomic control	Köttgen et al.(2013)^24^: a meta-analysis of 48 GWA studies for circulating serum urate levels (mg/dl) in 110,347 participants of European ancestry in the Global Urate Genetics Consortium (GUGC). The studies included in the meta-analysis were adjusted for age, sex, and study-specific covariates, where applicable (e.g., principal components and study center)
Obesity on HDL	Berndt et al*.* (2013)^23^	Willer et al*.* (2013)^25^: age-, sex-, and population-structure-adjusted GWA study of circulating HDL levels (mg/dl) in up to 187,167 individuals (largely of European ancestry). Blood lipid levels were measured after > 8 h of fasting, and those on lipid-lowering medications excluded
Obesity on LDL	Berndt et al*.* (2013)^23^	Willer et al*.* (2013)^25^: age-, sex-, and population-structure-adjusted GWA study of circulating LDL levels (mg/dl) in up to 173,082 individuals (largely of European ancestry). Blood lipid levels were measured after > 8 h of fasting, and those on lipid-lowering medications were excluded. LDL was directly measured in 24% of subjects and estimated with the Friedewald formula in the rest
Obesity on triglycerides	Berndt et al*.* (2013)^23^	Willer et al*.* (2013)^25^: age-, sex-, and population-structure-adjusted GWA study of circulating triglyceride levels (mg/dl) in up to 177,861 individuals (largely of European ancestry). Blood lipid levels were measured after > 8 h of fasting, and those on lipid-lowering medications excluded
HDL on urate	Willer et al*.* (2013)^25^	Köttgen et al.(2013)^24^
Triglycerides on urate	Willer et al*.* (2013)^25^	Köttgen et al.(2013)^24^
Urate on gout	Köttgen et al.(2013)^24^	GWA study of self-reported gout performed by the Medical Research Council-Integrative Epidemiology Unit (MRC-IEU) staff, using PHESANT-derived^26^ UK Biobank data^27,28^ (UK Biobank data field 20,002). It was adjusted for sex and genotyping chip. K-means cluster analysis for European ancestry was done (first four principal components, as provided by the UK Biobank)^29^. It contained 462,933 participants, largely of European descent^30^,, of which 6,542 with gout
Obesity on gout	Berndt et al*.* (2013)^23^	MRC-IEU GWA study of self-reported gout^27,28^

### Overview of designs

**Figure 1:**
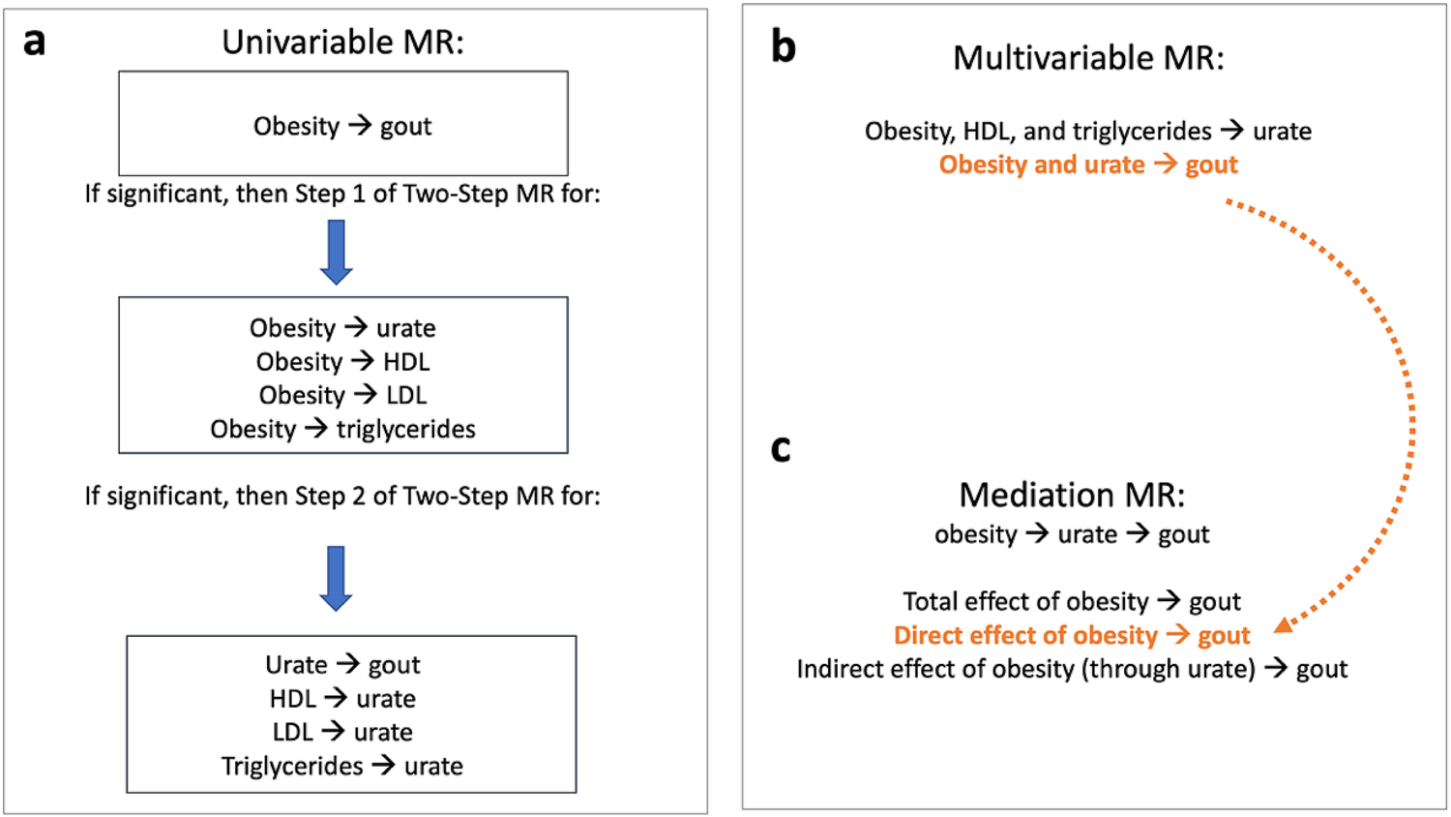
Overview of MR approaches used in the present study: univariable, two-step, multivariable, and mediation MR. (**a**) Flowchart of possible univariable and two-step MR models showing that only significant models from the Step 1 of two-Step MR are taken forward for Step 2 of two-step MR. (**b**) List of multivariable MR models. “Obesity and urate gout” was performed to obtain the direct effect of obesity on gout when accounting for urate and was used in the mediation analysis, as indicated by the orange coloring and arrow. (**c**) Mediation MR of obesity on gout with urate as the mediator.

We utilized a variety of two-sample MR designs (Fig. 1).

In addition to univariable MR (one exposure and one outcome), we performed two-step, multivariable, and mediation MR. We will now briefly explain the latter three.

Two-*step* MR (not to be confused with two-*sample* MR, which refers to the data sources) refers to two MR analyses done sequentially, such that the outcome variable of the first MR analysis is the exposure variable for the second MR analysis.

The goal is to take the MR procedure a step beyond knowledge of whether two variables are causally related and to infer a possible mechanism.

Here we are interested in knowing whether obesity impacts gout through causing changes to urate levels. The proposed mechanism, therefore, is a pathway from obesity to gout through urate. To explore this, we first instrumented obesity and tested the impact of obesity on urate levels (with a univariable MR analysis of obesity on urate). This is the first “step” in this two-step MR. For the second “step”, we instrumented urate levels and tested the impact of more urate on gout (univariable MR of urate on gout). The reasoning is this: If obesity influences urate and urate influences gout, then urate possibly mediates the relationship between obesity and gout. “Mediation” connotes the presence of a putative mechanism. Two additional two-step MR analyses were likewise performed:

- (1) Obesity on HDL and (2) HDL on urate
- (2) Obesity on triglycerides and (2) triglycerides on urate

Multivariable MR includes more than one independent variable in the MR model. It permits statistical adjustment, like multivariable regression and provides the direct effect of an exposure when accounting for the other variables in the model. 

MR can borrow and incorporate well-established techniques from observational epidemiology. One of these is mediation analysis. MR mediation analysis includes a combination of univariable and multivariable MR approaches. Two-step MR and mediation analysis are complementary approaches for testing pathways/mechanisms. Here we complement the two-step MR of obesity on urate and urate on gout with a mediation analysis to determine the indirect effect of obesity that occurs through obesity’s impact on urate.

Three parameters are usually estimated in epidemiologic mediation analyses: (i) the total effect (the overall effect of an exposure on an outcome through all potential pathways), (ii) the direct effect (the effect of the exposure on an outcome not due to a mediator of interest), and (iii) the indirect effect (the effect of exposure on the outcome due to the mediator)^31^.

In the context of MR, mediation analysis can be done by generating the total effect with univariate MR (e.g., the effect of obesity on gout). The direct effect can be obtained with multivariate MR (e.g., the effect of obesity on gout when adjusting for urate). The indirect effect (e.g., the effect on risk for gout that occurs through the mediator, i.e., increased urate levels) can be calculated by either subtracting the direct effect from the total effect or by multiplying the effect of the exposure on the mediator by the effect of the mediator on the outcome, using the product of coefficients approach^31^. We used the product of coefficients approach.

Typically, alongside the total, direct, and indirect effects, a proportion mediated can also be calculated, except when there is evidence for “inconsistent mediation” (a scenario in which the direct and indirect effects are in the opposite directions)^32^. The present MR mediation results (discussed under Results) are an example of inconsistent mediation (i.e., the direct effect of obesity on gout and the indirect effect of obesity on gout are in opposite directions). Thus, the proportion mediated is not calculated.

### Instrument construction

For all the MR approaches, an “instrument” had to be constructed. The summary statistics for the SNPs strongly associated with the exposure traits are the instruments. As mentioned above, the reader can view these in Supplemental tables 10–12 and 14–18.

Independent (those not in LD; R^2^ < 0.001) SNPs associated at genome-wide significance (*P* < 5 × 10^–8^) with exposure traits were extracted from GWA studies for the exposures in this analysis. SNP selection was done within the “TwoSampleMR” package^17,33^. The summary statistics for the exposure-associated SNPs were then extracted from each of the outcome GWA studies. SNP-exposure and SNP-outcome associations were harmonized and combined with the inverse variance-weighted (IVW) method using first-order weights.

### Sensitivity analyses

The IVW estimator can be biased in the presence of unwanted pleiotropy^34^. To address this, we included a battery of sensitivity estimators, including MR-Egger regression, weighted median, and weighted mode MR methods. The directions and magnitudes of their effect estimates were compared with those of the IVW^35^. If the IVW and sensitivity estimators comport, this provides a qualitative screen against pleiotropy; since the various MR estimators make different assumptions, in the presence of substantial pleiotropy, their estimates are unlikely to align^34,36,37^.

Of note: MR-Egger regression, in addition to providing a (sensitivity) estimate for a causal effect, includes a formal test for directional pleiotropy (the “MR-Egger intercept test”). When the MR-Egger intercept is consistent with 0 (or 1 when exponentiated; *P* > 0.05), this provides some evidence against pleiotropy in the IVW estimate.

Certain assumptions for MR-Egger regression must hold, however, for this to be true. MR-Egger requires there be negligible measurement error in the SNP-exposure estimates^38^. Violations to this can dilute the MR-Egger estimate and make the MR-Egger intercept test a potential false positive. *I*^2^ statistics are used to check for this. For example, when *I*^2^ statistics are close to 90%, this suggests about 10% possible bias due to measurement error. Simulation extrapolation (SIMEX) correction can be performed if the bias is much greater than 10%, and is recommended in such cases^34,38,39^. We calculated *I*^2^ statistics for the univariate models, which suggested minimal bias due to this problem (SIMEX correction was also run nonetheless; see Supplementary tables 1–3 and 5–9). SIMEX correction was implemented using the “mreggersimex” command within Stata using the package “mrrobust” package^34^. If there was evidence for attenuation in the MR-Egger estimate, SIMEX correction could have provided a less-biased estimate.

Lastly, a final measure was taken to ward against pleiotropy. Heterogeneity in the MR effect estimates for SNPs can indicate unwanted pleiotropy. As such, potential outlier SNPs were removed, using IVW “RadialMR” regression^40^ for all univariable models. Outliers were determined with respect to heterogeneity quantified by Cochran’s *Q*-statistic. A significance threshold of 0.05 was set for identifying outliers, and first-order weights were specified. Due to removing outliers, the various MR tests that have the same exposure (i.e., obesity on urate, obesity on triglycerides, obesity on HDL, etc.) may contain differing numbers of SNPs (Table 2 contains the number of SNP instruments used for each univariable test). All instrumental variables included in this analysis have Cochrane’s *Q*-statistic *P*-values indicating no evidence for heterogeneity between SNPs^41^^.^ Heterogeneity statistics and outlier SNPs that were removed are available in Supplementary tables 1–9. (Forest plots of results and scatter plots for the univariable tests are available in the Supplementary figures file).

**Table 2.** Univariable MR results and sensitivity analyses.

Test (no. SNPs)	R^2^	*F*	IVW			MR-Egger			MR-Egger intercept			Weighted median			Weighted mode		
			Beta or OR	95% CI	*P*	Beta or OR (*I*^2^)	95% CI	*P*	Beta or OR	95% CI	*P*	Beta or OR	95% CI	*P*	Beta or OR	95% CI	*P*
Obesity on HDL (14)	0.07	506	Beta − 0.083	− 0.101, − 0.065	2.5E−19*	Beta − 0.075 (90)	− 0.123, − 0.027	9.7E−03	Beta − 0.001	− 0.006, 0.004	7.4E−01	Beta − 0.077	− 0.102, − 0.052	1.7E−09	Beta − 0.077	− 0.103, − 0.050	6.9E−05
Obesity on LDL (13)	0.07	534	Beta − 0.011	− 0.030, 0.008	2.6E−01	Beta − 0.020 (90)	− 0.072, 0.034	4.8E−01	Beta 0.001	− 0.005, 0.007	7.3E−01	Beta 0.002	− 0.024, 0.030	8.7E−01	Beta 0.001	− 0.031, 0.034	9.6E−01
Obesity on TRI (15)	0.08	611	Beta 0.082	0.065, 0.099	1.2E−21*	Beta 0.068 (90)	0.021, 0.115	1.5E−02	Beta 0.002	− 0.004, 0.007	5.4E−01	Beta 0.081	0.054, 0.107	1.9E−09	Beta 0.078	0.049, 0.107	1.3E−04
HDL on urate (64)	0.05	81	Beta − 0.109	− 0.148, − 0.071	2.7E−08*	Beta − 0.071 (97)	− 0.149, 0.006	7.4E−02	Beta − 0.002	− 0.005, 0.001	2.7E−01	Beta − 0.122	− 0.180, − 0.064	3.8E−05	Beta − 0.115	− 0.182, − 0.049	1.1E−03
TRI on urate (35)	0.02	67	Beta 0.198	0.146, 0.251	8.9E−14*	Beta 0.114 (97)	0.021, 0.207	2.1E−02	Beta 0.004	0.000, 0.008	3.8E−02	Beta 0.174	0.092, 0.256	2.7E−05	Beta 0.149	0.067, 0.232	1.1E−03
Obesity on gout (13)	0.07	525	OR 1.003	1.001, 1.004	1.3E−04*	OR 1.003 (91)	0.999, 1.006	1.8E−01	OR 1.000	1.000, 1.000	9.9E−01	OR 1.002	1.000, 1.004	1.9E−02	OR 1.002	1.000, 1.004	1.1E−01
Obesity on urate (13)	0.06	514	Beta 0.127	0.098, 0.157	1.2E−17*	Beta 0.163 (91)	0.086, 0.239	1.6E−03	Beta − 0.004	− 0.013, 0.004	3.5E−01	Beta 0.114	0.073, 0.155	6.2E−08	Beta 0.120	0.072, 0.168	3.6E−04
Urate on gout (16)	NA	NA	OR 1.030	1.028, 1.032	1.1E−130*	OR 1.034 (88)	1.026, 1.042	9.2E−07	OR 1.000	0.999, 1.000	3.2E−01	OR 1.031	1.027, 1.034	1.4E−55	OR 1.033	1.027, 1.038	9.6E−09

*R*^2^ = is the proportion of variability in the exposure explained by the SNPs; *F* = *F*-statistic (*F* > 10 indicates adequate power); OR = odds ratio; CI = confidence interval. HDL = high-density lipoprotein cholesterol; LDL = low-density lipoprotein cholesterol; TRI = triglycerides. The MR-Egger intercept is highlighted in grey because its interpretation is different than the IVW and sensitivity estimators: *P*-value < 0.05 for the MR-Egger intercept suggests pleiotropy in the IVW estimator. *IVW *P*-value (*P* < 0.05/10 = 0.005) is considered significant for the IVW results. The directions and the magnitudes of the MR-Egger, weighted median, and weighted mode estimates are qualitatively compared with the direction and magnitude of the IVW estimate.

### Multiple testing

Altogether there were eight univariable MR tests (including the three two-step MRs), one multivariable MR, and one formal MR mediation analysis, making 10 tests. To account for multiple testing, a Bonferroni threshold for stringent evidence was set to *P* < 0.05/10 = 0.005 for the IVW, multivariable MR, and mediation analysis results. *P* < 0.05 was considered nominally significant and suggestive. In Tables 2, 3, 4, IVW results with *P*-values < 0.005 are marked with an asterisk.

**Table 3:** Multivariable MR of obesity, HDL, and triglycerides on urate.

	SNP	Beta	Lower 95% CI	Upper 95% CI	*P*-value
Obesity	10	0.104	0.071	0.136	4.0E−10*
HDL	62	− 0.084	− 0.130	− 0.039	2.8E−04*
Triglycerides	27	0.068	0.005	0.132	3.6E−02

HDL: high-density lipoprotein cholesterol; CI: confidence interval.

**Table 4:** Total, direct, and indirect effects of obesity on gout, with urate as the mediator.

	OR	Lower 95% CI	Upper 95% CI	*P*-value
Total effect of obesity on gout	1.003	1.001	1.004	8.2E−09*
Direct effect of obesity on gout	0.999	0.997	1.000	1.9E−01
Indirect effect (through urate)	1.004	1.003	1.005	2.5E−14*

OR: odds ratio; CI: confidence interval.

### Software

The IVW and sensitivity estimations were performed in R version 3.6.2 with the “TwoSampleMR” package^17,33^. The multivariable MR analysis was performed using the “mv_multiple” function for generating IVW estimates, also within the “TwoSampleMR” package, after clumping for LD (PLINK clumping method with 10,000 kilobase [kb] window; R^2^ of 0.001), harmonizing^21^, and removing outlier SNPs (identified with “RadialMR”), which had been removed in the univariate analyses. The standard error for the indirect effect was approximated with the delta method^32^. Genetic correlations were calculated using the ldsc software (available at http://www.github.com/bulik/ldsc)42.

## Results

### Univariable MR tests

#### Obesity on HDL

Obesity decreased HDL levels (beta = − 0.083; 95% confidence interval (CI) = − 0.101, − 0.065; *P*-value = 2.5E−19). The sensitivity estimators (MR-Egger, weighted median and weighted mode) aligned in their directions and magnitudes of effects (MR-Egger [− 0.075], weighed median [− 0.077], and weighted mode [− 0.077]), providing no evidence for pleiotropy (see Table 2).

#### Obesity on LDL

There was a lack of evidence that obesity (conceived of as a risk factor for higher LDL levels) impacts LDL (beta = − 0.011; 95% CI = − 0.030, 0.008; *P*-value = 2.6E−01). The sensitivity estimators were discrepant across their directions of effect (MR-Egger [− 0.020], weighted median [0.002], and weighted mode [0.001]), which is suggestive of pleiotropic distortion. Due to this, LDL was not taken forward in the multivariable analyses.

#### Obesity on triglycerides

Obesity (conceived of as a risk factor for higher triglycerides) increased triglyceride levels (beta = 0.082; 95% CI = 0.065, 0.099; *P*-value = 1.2E−21). The sensitivity estimators aligned (MR-Egger [0.068], weighted median [0.081], and weighted mode [0.078]).

#### HDL on urate

There was strong evidence that higher HDL (conceived of as a protective factor against higher urate levels) decreased urate levels (beta estimate per unit higher HDL level (mg/dl) = − 0.109; 95% CI = − 0.148, − 0.071; *P*-value = 2.7E−08). The sensitivity estimators aligned (MR-Egger [− 0.071], weighted median [− 0.122], and weighted mode [− 0.115]).

#### Triglycerides on urate

There was strong evidence that higher triglycerides (conceived of as a risk factor for higher urate levels) increased urate levels (beta estimate per unit higher triglyceride level (mg/dl) = 0.198; 95% CI = 0.146, 0.251; P-value = 8.9E−14). The sensitivity estimators aligned in the direction of their effects but were somewhat discrepant in their magnitudes (MR-Egger [0.114], weighted median [0.174], and weighted mode [0.149]). The results for the MR-Egger intercept test reveal potential pleiotropy: MR-Egger intercept: 0.004; 95% CI = 0.000, 0.008; *P*-value = 3.8E−02.

#### Obesity on gout

Obesity (conceived of as a risk factor for gout) causally impacted the risk for gout, driving it higher (odds ratio, OR = 1.003; 95% CI = 1.001, 1.004; *P*-value = 1.3E−04). The sensitivity estimators aligned (MR-Egger [1.003], weighted median [1.002], and weighted mode [1.002]).

#### Obesity on urate levels

Obesity (conceived of as a risk factor for higher urate levels) raised urate levels (beta = 0.127; 95% CI = 0.098, 0.157; *P*-value = 1.2E−17). The sensitivity estimators aligned in the direction of their effects but varied somewhat in their magnitudes: MR-Egger [0.163], weighted median [0.114], and weighted mode [0.120]).

#### Urate on gout

Higher urate levels (conceived of as a risk factor for gout) increased the odds of gout (OR = 1.030; 95% CI = 1.028, 1.032; *P*-value = 1.1E−130). The sensitivity estimators aligned (MR-Egger [1.034], weighted median [1.031], and weighted mode [1.033]).

**Figure 2:**
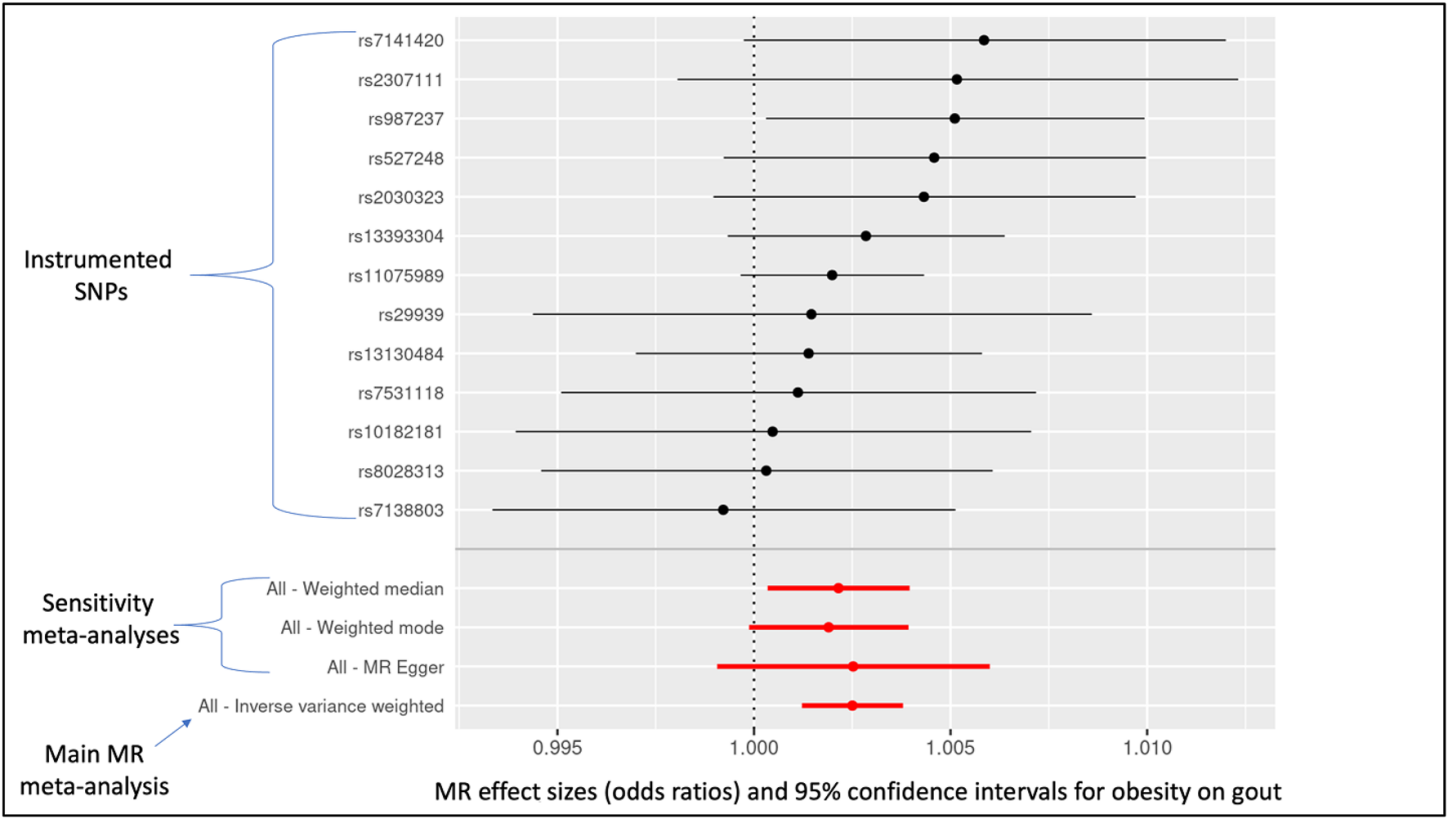
Individual-SNP and meta-analytic MR effect estimates (odds ratios) and 95% confidence intervals for the effect of obesity on gout. Weighted median, weighted mode, and MR-Egger are sensitivity meta-analyses. The inverse-variance weighted (IVW) result is the main MR test.

**Figure 3:**
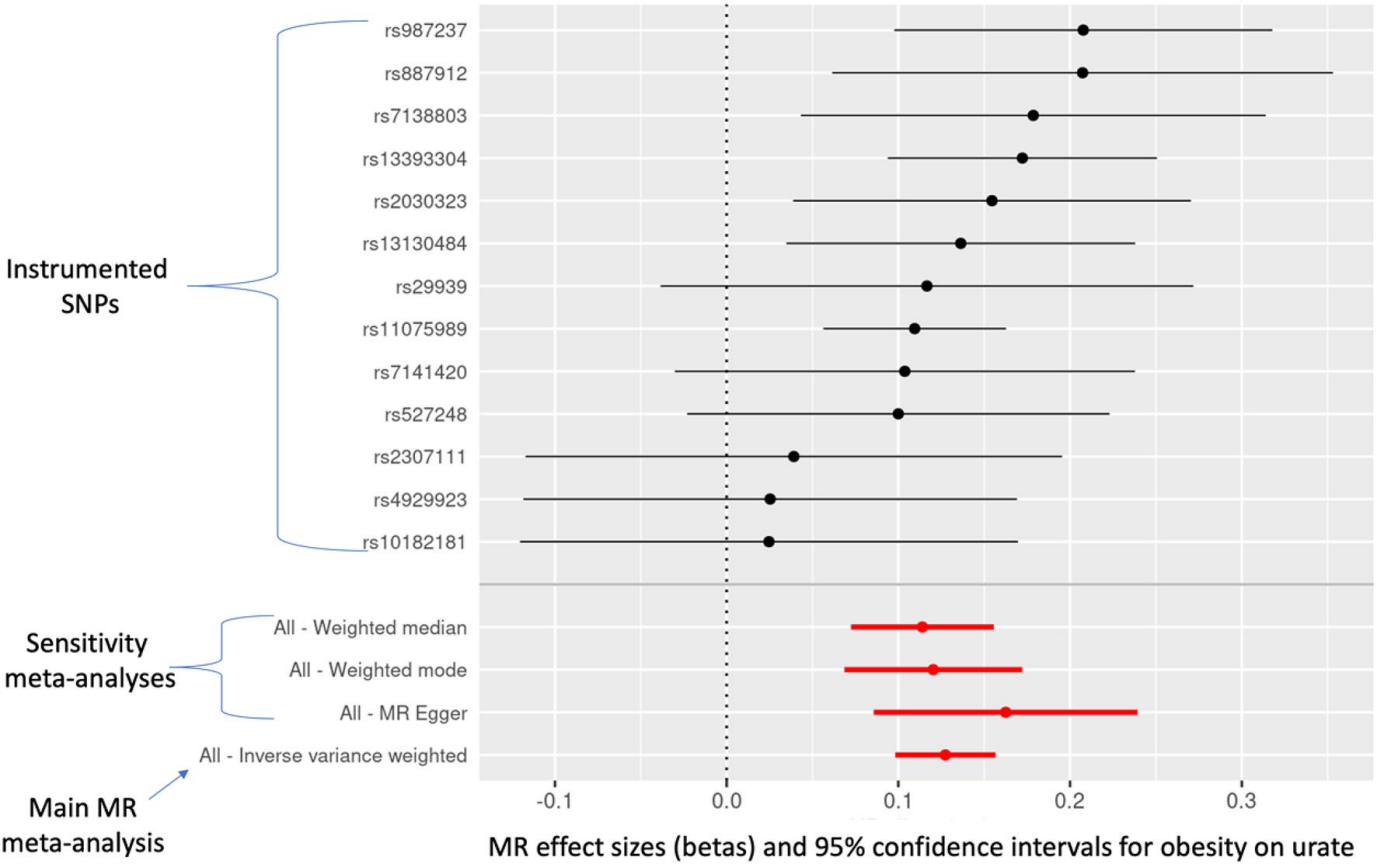
Individual-SNP and meta-analytic MR effect estimates and 95% confidence intervals for the effect of obesity on urate. Weighted median, weighted mode, and MR-Egger are sensitivity meta-analyses. The inverse-variance weighted (IVW) result is the main MR test.

**Figure 4:**
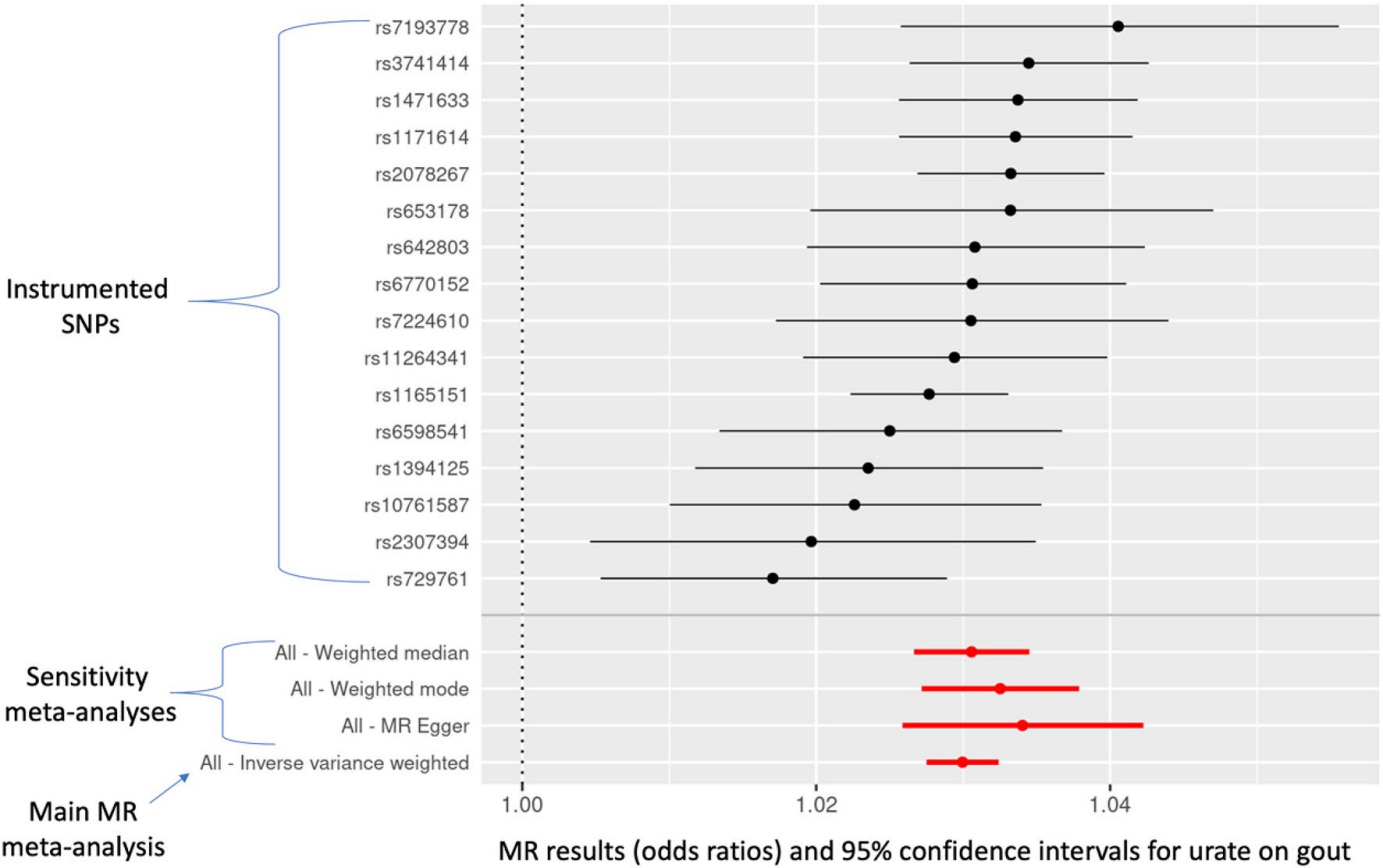
Individual-SNP and meta-analytic MR effect estimates (odds ratios) and 95% confidence intervals for the effect of urate on gout. Weighted median, weighted mode, and MR-Egger are sensitivity meta-analyses. The inverse-variance weighted (IVW) result is the main MR test.

These results for these last three reported tests are displayed in Figs. 2, 3, 4.

Although not the focus of the present study, we performed an MR analysis of urate on T2D, a study that has been previously done^3^. We did this to replicate the previous finding. The MR of urate on T2D was null (OR = 0.945; 95% CI = 0.832, 1.074; *P* = 0.385), consistent with what was observed by Sluijs et al. (2015): OR = 0.99; 95% CI = 0.92, 1.06.

### Multivariable MR

The multivariable MR of obesity, HD, and triglyceride on urate revealed strong evidence that obesity and HDL directly impacted urate levels, with obesity leading to higher urate (beta = 0.104; 95% CI 0.071, 0.136; *P*-value = 4.0E−10) and HDL decreasing urate (beta = − 0.084; 95% CI − 0.130, − 0.039; *P*-value = 2.8E−04). There was suggestive evidence that higher triglycerides also increased urate levels (beta = 0.068; 95% 0.005, 0.132; *P*-value = 3.6E− 02). See Table 3.

### MR mediation analysis

The formal mediation analysis of obesity on gout with urate as a mediator revealed that nearly all the effect of obesity on gout is mediated through urate. The reason for this is that the direct effect of obesity on gout is essentially null with the confidence interval including 1 with rounding (OR = 0.999; 95% CI 0.997, 1.000; *P* = 0.038) whereas the total and indirect effects are not (Table 4). (The upper limit for the confidence interval is slightly less than 1 prior to rounding, explaining what looks like, but is not, a discrepancy between the *P*-value and the confidence interval. The message is the same: the direct effect of obesity on gout is trivial).

### Genetic correlations

SNP heritability, the proportion of variation in the traits explained by additive SNPs (${h}_{g}^{2}$), was > 0 (observed scale) for all traits for which genetic correlations were calculated (Supplementary table 22): obesity (${h}_{g}^{2}$=0.17; 95% CI = 0.15, 0.18); urate (${h}_{g}^{2}$=0.17; 95% CI = 0.03, 0.31); HDL (${h}_{g}^{2}$=0.25; 95% CI = 0.17, 0.32); LDL (${h}_{g}^{2}$=0.21; 95% CI = 0.11, 0.30); triglycerides (${h}_{g}^{2}$=0.27; 95% CI = 0.16, 0.38); gout (${h}_{g}^{2}$=0.02; 95% CI = 0.01, 0.04).

Obesity was negatively genetically correlated with HDL (*r*_*g*_ = − 0.28; *P*-value = 5.2E−24). The correlation for obesity and LDL was null (*r*_*g*_ = 0.03; *P*-value = 0.369). Obesity was positively genetically correlated with triglycerides (*r*_*g*_ = 0.23; *P*-value = 8.8E−12), urate (*r*_*g*_ = 0.25; *P*-value = 0.001), and gout (*r*_*g*_ = 0.23; *P*-value = 2.7E−05). Urate was negatively genetically correlated with HDL (*r*_*g*_ = −0.21; *P*-value = 9.8E−05) and positively genetically correlated with gout (*r*_*g*_ = 0.88; *P*-value = 1.68E−55). The genetic correlation between LDL and urate was null (*r*_*g*_ = 0.06; *P*-value = 0.084). Triglycerides and urate were positively correlated (*r*_*g*_ = 0.29; *P*-value = 0.001). See Supplementary table 22 to view these findings in a table.

## Discussion

To summarize our results, we found that obesity increased urate and triglycerides and decreased HDL. Higher triglycerides increased urate, and higher HDL decreased urate. Higher urate and obesity increased risk for gout. The mediation MR of obesity on gout with urate as a mediator revealed, however, that all the effect of obesity on gout is mediated through urate. The impact of obesity on LDL was null. The multivariable MR of obesity, HDL, and triglycerides on urate revealed that obesity influences urate even when accounting for HDL and triglycerides.

Genetic correlations for the pairs of traits were concordant with our MR findings. Both the MR of obesity on LDL and the genetic correlation for obesity and LDL were null. This provides evidence against a causal relationship between obesity and LDL. Similarly, the following pairs comport: the MR of obesity on HDL and the genetic correlation for obesity and HDL suggest obesity decreases HDL; the MR of obesity on triglycerides and the genetic correlation between obesity and triglycerides suggest that obesity increases triglycerides; the MR of HDL on urate and the genetic correlation between HDL and urate suggest that HDL lowers urate; the MR of obesity on gout and the genetic correlation between obesity and gout suggest that obesity causes gout; the MR of urate on gout and the genetic correlation of urate and gout suggest that urate causes gout.

Obesity impacting gout through urate confirms what has been observed in a robust cannon of epidemiologic literature^9^. MR results can conflict with epidemiologic findings, though, and it was reasonable to think that MR might not replicate the knowledge from the observational studies. Gout exists in a tangled web of metabolic and inflammatory phenotypes, and previous epidemiologic and MR studies have been discrepant with regard to urate’s impact on T2D: the epidemiologic data suggest that urate increases risk for T2D, but MR studies (including the analysis we conducted, available in Supplementary table 4) imply the epidemiologic association is confounded or reverse caused^3^. In contrast, our findings for the relationship between urate and gout are an example of MR triangulating (confirming) the previous epidemiologic literature for urate and gout. This helps build a causal story, since causality is not determined from single studies but is a “case” assembled from layers of evidence.

Importantly, from the formal MR mediation analysis, our results suggest that all the effect of obesity on gout occurred via obesity’s impact on urate (an instance of “inconsistent mediation”). Obesity’s effect on urate, moreover, appears to be occurring by decreasing HDL levels and increasing triglycerides. However, obesity also had a direct effect on urate when accounting for both HDL and triglycerides. This suggests that the mechanisms by which obesity impacts urate, and thus gout, are not fully captured by obesity’s impacts on HDL and triglycerides. There may be other intermediate traits which obesity influences, which in turn influence urate and gout. Our data provided evidence that this possible third trait is not LDL, since the impact of obesity on LDL did not appear to be causal.

One intriguing avenue for future research would be to consider molecular mechanisms. For instance, it is possible that obesity dysregulates microRNAs (miRNAs; short, single-stranded RNAs that post-transcriptionally regulate gene expression) that influence urate or traits that influence it. Recently, Bohatá et al*.* (2021) found five upregulated miRNAs in plasma of patients with hyperuricemia and gout^43^. Though the direction of causality cannot be determined from their study, Bohatá et al.’s findings suggest involvement of miRNAs in the development of gout, either etiologically or as a biomarker. Likewise, Nikpay et al*.* (2019) performed a GWA study of circulating-miRNAs, which generated miRNA expression quantitative trait loci (mirQTL). They used the mirQTLs to instrument miRNAs and performed two-sample MR of miRNA expression on cardiometabolic traits^44^. They observed that higher plasma levels of miR-199a were associated with lower levels of LDL and total cholesterol. This lends credibility to the idea that miRNAs might influence other lipids. Since their GWA study was small (only 710 people), larger mirQTL studies may unveil additional mirQTLs, possibly ones that influence HDL. Future MR studies could be done to examine whether miRNAs influence urate and gout. 

Our results comport with previous MR studies of cardiovascular traits, urate, and gout. Larsson, Burgess, and Michaëlsson (2018) found that genetically higher BMI, though not waist-to-hip ratio adjusted for BMI, was positively associated with both urate and gout^45^. Similarly, Lyngdoh et al*.* (2012), who performed a bidirectional MR study, found evidence that higher adiposity caused higher urate^46^. Because our anthropomorphic measure is “obesity” not “BMI” or “adiposity”, our results complement theirs and support the common-sense understanding that the three measures are connected. Our findings differ some from Holmes et al*.* (2014), whose genetic instrument for higher BMI causally lowered LDL^47^. Although we also observed that obesity lowered LDL, it was not significant, and the sensitivity estimators indicated pleiotropy. Of note is that Holmes’ MR result contradicted the epidemiological data that suggested that higher BMI increased LDL^47^.

Major strengths of our analyses were the use of multiple large GWA studies, capitalizing on their large sample sizes, and the various sensitivity tests we performed to investigate unwanted pleiotropy. The only tests that showed evidence for possible unwanted pleiotropy were those for the impact of obesity on LDL and triglycerides on urate. We did not see evidence for horizontal pleiotropy for the relationships between obesity, urate, and gout.

Our study has limitations. The primary one is inherent to MR: although we employed multiple sensitivity estimators to check for it, unwanted pleiotropy can never be ruled out in any MR study^48^. A second limitation is similar to the first: although it is a strength that we used two methods that protect against environmental confounding, MR and genetic correlation can both suffer from genetic confounding^42,48^. A third limitation is that we were unable to examine sex-specific effects of obesity on urate or gout. Globally, the prevalence of obesity is higher for women (15%) than men (11%)^49^, yet gout is more common in men^50^. A fourth limitation is that our findings are only applicable to those of European ancestry.

Taken as a whole, our results reveal several key points, some previously understood and others less apparent from prior research. While obesity itself is a complex phenotype with various convergent pathways conspiring to produce it, the downstream effects of obesity are also complex and include a variety of deleterious outcomes. Here, we sought to better understand possible pathways leading from obesity to the chronic and painful disorder of gout. When considered as the primary “exposure variable”, obesity appeared to operate antagonistically against protective factors (by lowering HDL), while exacerbating risk factors for gout (increasing urate). Our primary contribution was to reveal an apparent mediation effect whereby obesity raised urate levels and, through this, increased likelihood of gout. Indeed, all the effect of obesity on gout appeared to result from the rise in urate. Considered via a translational lens, our results suggest that management of urate should help to blunt the effects of obesity on the development of gout.

## Supplementary Information

Supplementary Figures.

Supplementary Tables.

## Data Availability

There was no human participation in this study. All data sources for summary statistics used in the present analyses are freely available through MR-Base (http://www.mrbase.org/17).
