# Supplementary material for: Using multiple Mendelian randomization approaches and genetic correlations to understand obesity, urate, and gout
Source: Sci Rep. 2021 Sep 7;11:17799. doi: 10.1038/s41598-021-97410-4 (PMC8423843; doi:10.1038/s41598-021-97410-4)

What multiple Mendelian randomization approaches and genetic correlations reveal about obesity and gout

Authors:

Charleen D. Adams[1*] and Brian B. Boutwell[2,3]

[1]Department of Environmental Health, Program in Molecular and Integrative Physiological Sciences, Harvard T.H. Chan School of Public Health, Boston, Massachusetts 02115, USA;[2] School of Applied Science, The University of Mississippi P.O. Box 1848, University, MS, 38677, USA; [3] John D. Bower School of Population Health, University of Mississippi Medical Center, Jackson, MS, 39216, USA

[*] Correspondence to:

Department of Environmental Health, Program in Molecular and Integrative Physiological Sciences, Harvard T.H. Chan School of Public Health, Boston, Massachusetts 02115, USA; Tel.: 626-841-3937; Email: cdadams@hsph.harvard.edu

Legend

Supplementary figure 1. Forest plot for MR of obesity on gout.
Supplementary figure 2. Scatter plot for MR of obesity on gout.
Supplementary figure 3. Forest plot for MR of obesity on urate.
Supplementary figure 4. Scatter plot for MR of obesity on urate.
Supplementary figure 5. Forest plot for MR of urate on gout.
Supplementary figure 6. Scatter plot for MR of urate on gout.
Supplementary figure 7. Forest plot for MR of urate on type 2 diabetes (T2D).
Supplementary figure 8. Scatter plot for MR of urate on type 2 diabetes (T2D).
Supplementary figure 9. Forest plot for MR of obesity on high-density lipoprotein cholesterol (HDL).
Supplementary figure 10. Scatter plot for MR of obesity on high-density lipoprotein cholesterol (HDL).
Supplementary figure 11. Forest plot for MR of obesity on low-density lipoprotein cholesterol (LDL).
Supplementary figure 12. Scatter plot for MR of obesity on low-density lipoprotein cholesterol (LDL).
Supplementary figure 13. Forest plot for MR of obesity on triglycerides.
Supplementary figure 14. Scatter plot for MR of obesity on triglycerides.
Supplementary figure 15. Forest plot for MR of high-density lipoprotein (HDL) on urate.
Supplementary figure 16. Scatter plot for MR of high-density lipoprotein (HDL) on urate.
Supplementary figure 17. Forest plot for MR of triglycerides on urate.
Supplementary figure 18. Scatter plot for MR of triglycerides on urate.

Supplementary figure 1. Forest plot for MR of obesity on gout.

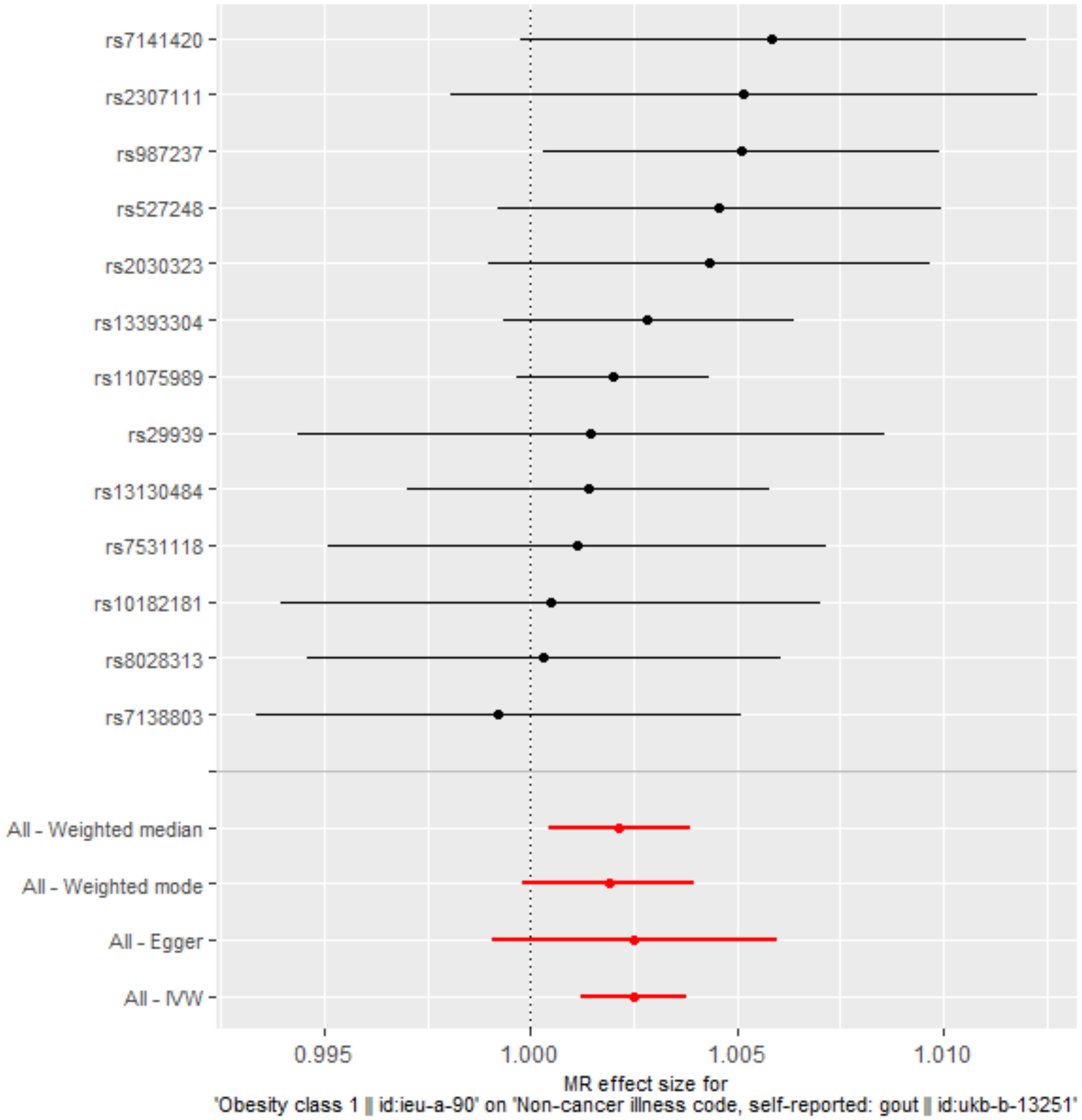

The y-axis contains the instrumental SNPs and meta-analyses of their Wald ratios: inverse-variance weighted (IVW; the main MR result) and sensitivity estimators (weighted median, weighted mode, and MR-Egger). The magnitude and direction of the effects of the sensitivity estimators are compared visually with those of the IVW. When the estimators align (as shown above), this is evidence against pleiotropy.

Supplementary figure 2. Scatter plot for MR of obesity on gout.

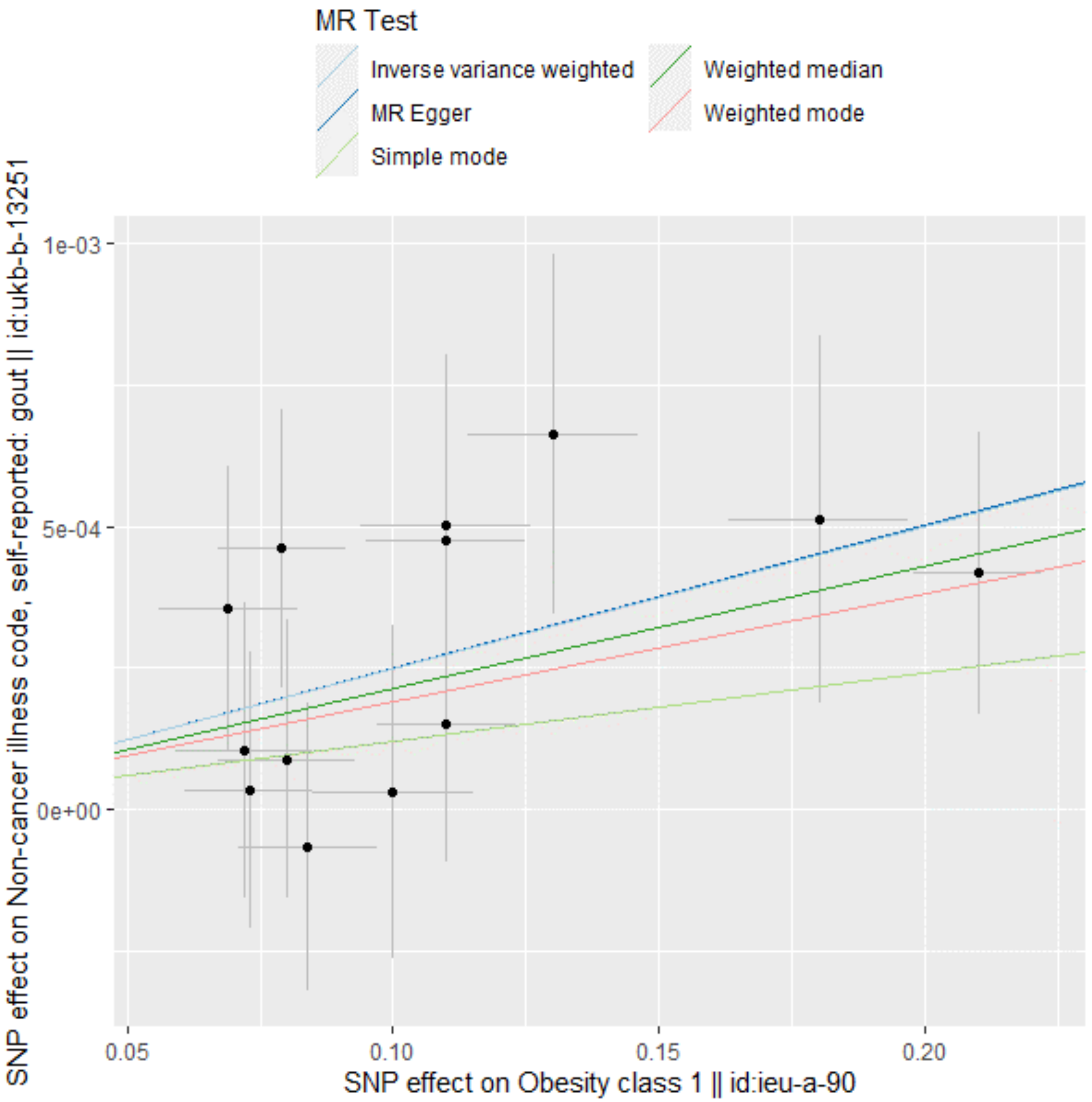

Supplementary figure 3. Forest plot for MR of obesity on urate.

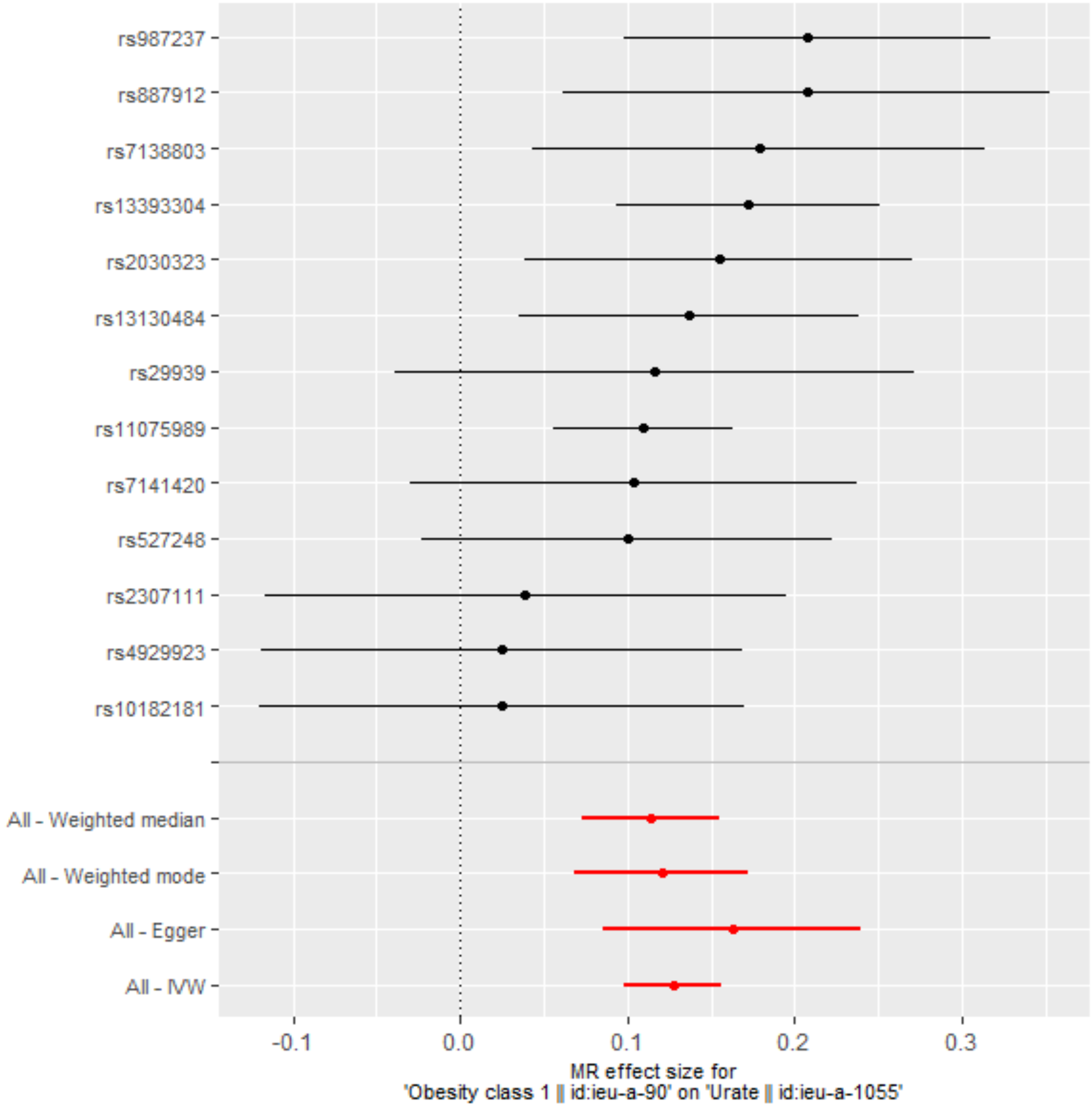

The y-axis contains the instrumental SNPs and meta-analyses of their Wald ratios: inverse-variance weighted (IVW; the main MR result) and sensitivity estimators (weighted median, weighted mode, and MR-Egger). The magnitude and direction of the effects of the sensitivity estimators are compared visually with those of the IVW. When the estimators align (as shown above), this is evidence against pleiotropy.

Supplementary figure 4. Scatter plot for MR of obesity on urate.

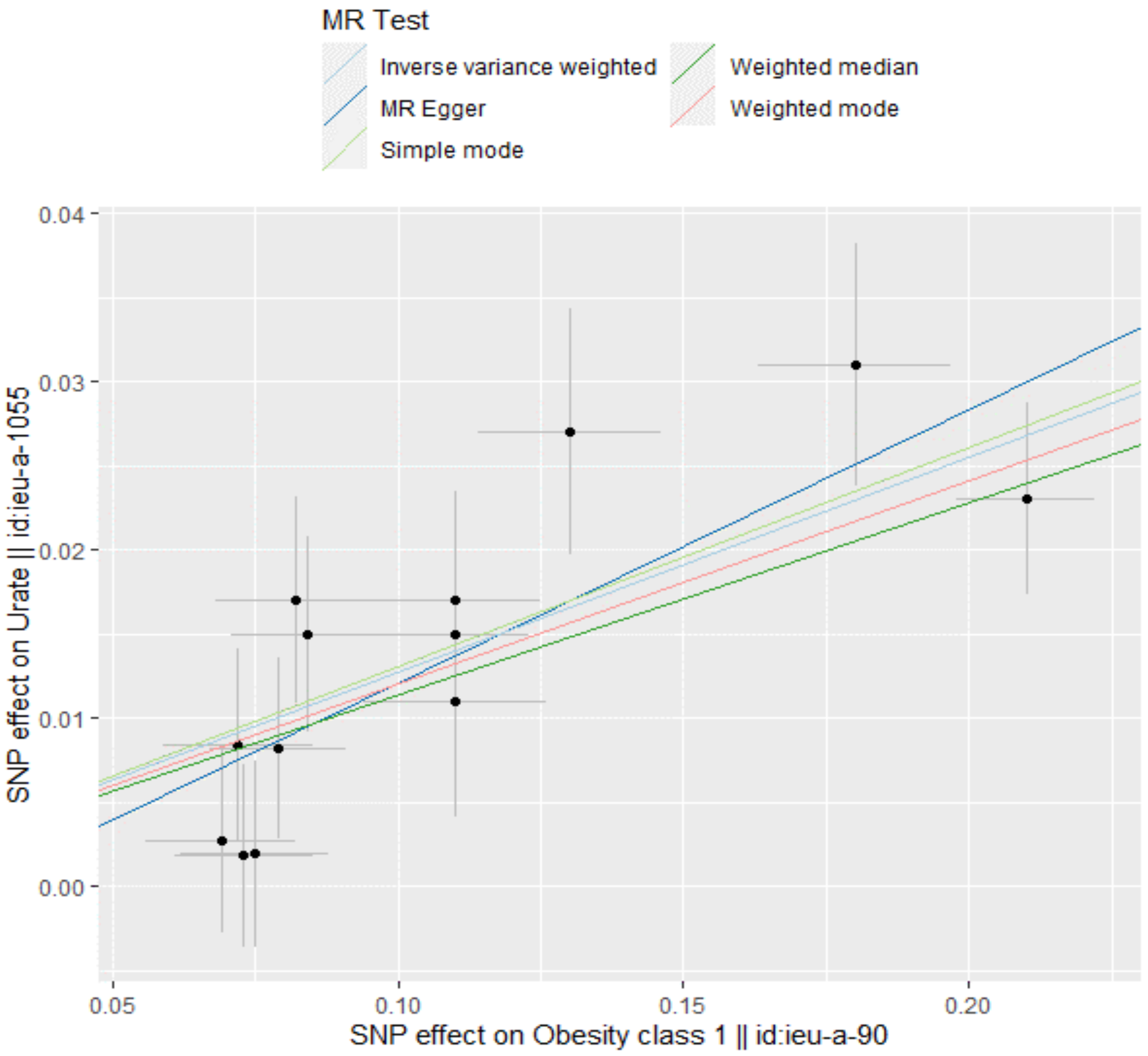

Supplementary figure 5. Forest plot for MR of urate on gout.

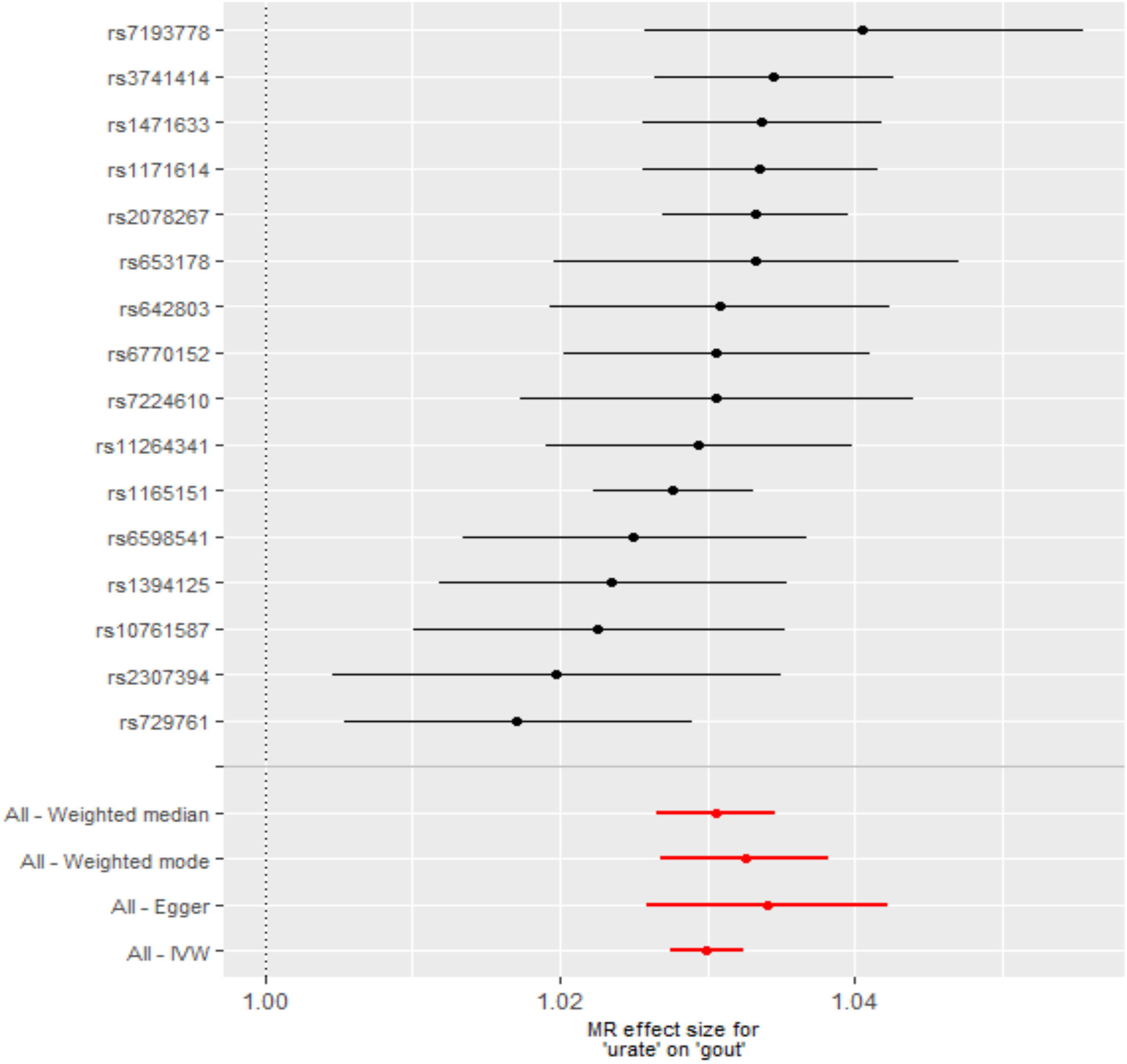

The y-axis contains the instrumental SNPs and meta-analyses of their Wald ratios: inverse-variance weighted (IVW; the main MR result) and sensitivity estimators (weighted median, weighted mode, and MR-Egger). The magnitude and direction of the effects of the sensitivity estimators are compared visually with those of the IVW. When the estimators align (as shown above), this is evidence against pleiotropy.

Supplementary figure 6. Scatter plot for MR of urate on gout.

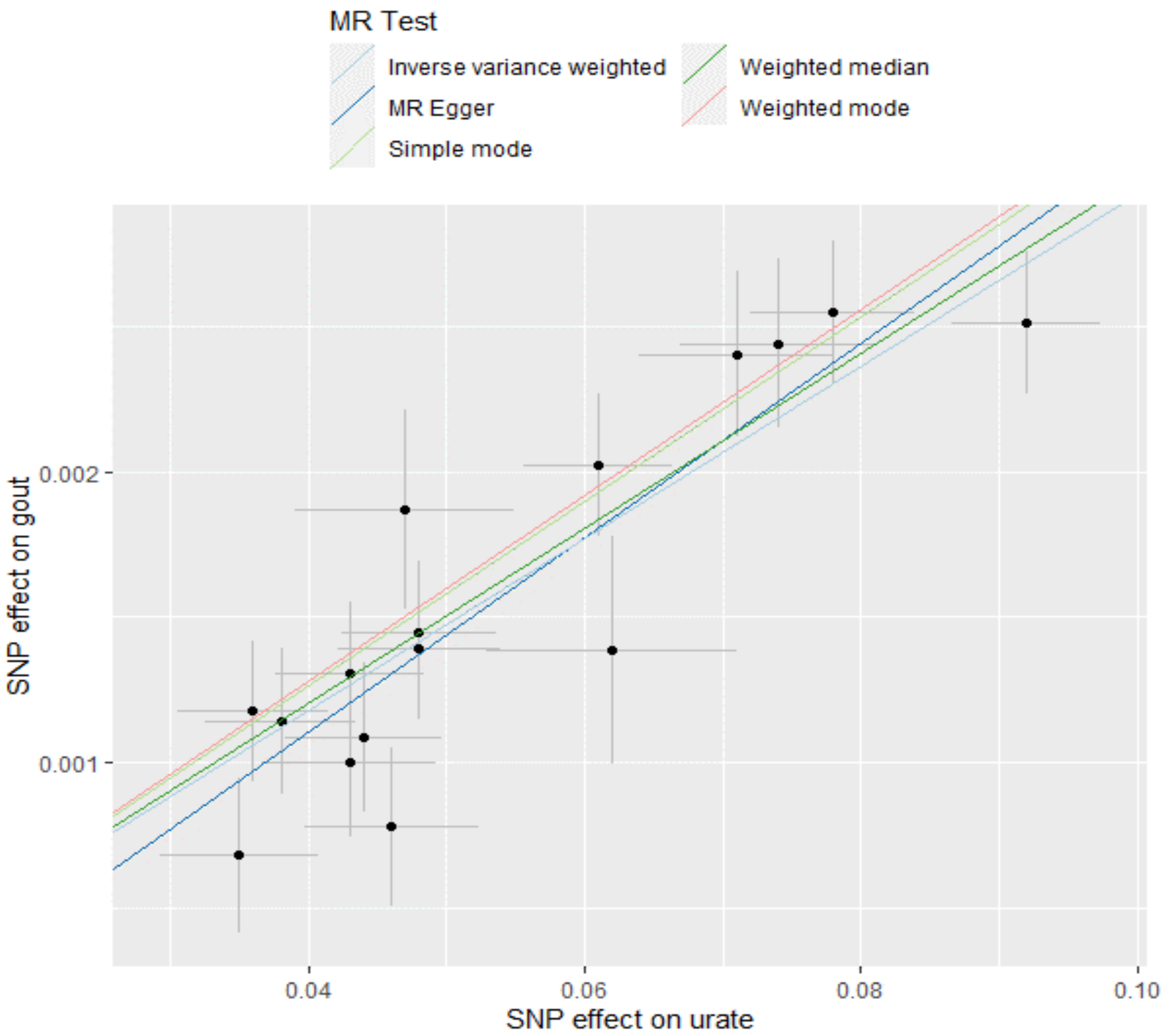

Supplementary figure 7. Forest plot for MR of urate on type 2 diabetes (T2D).

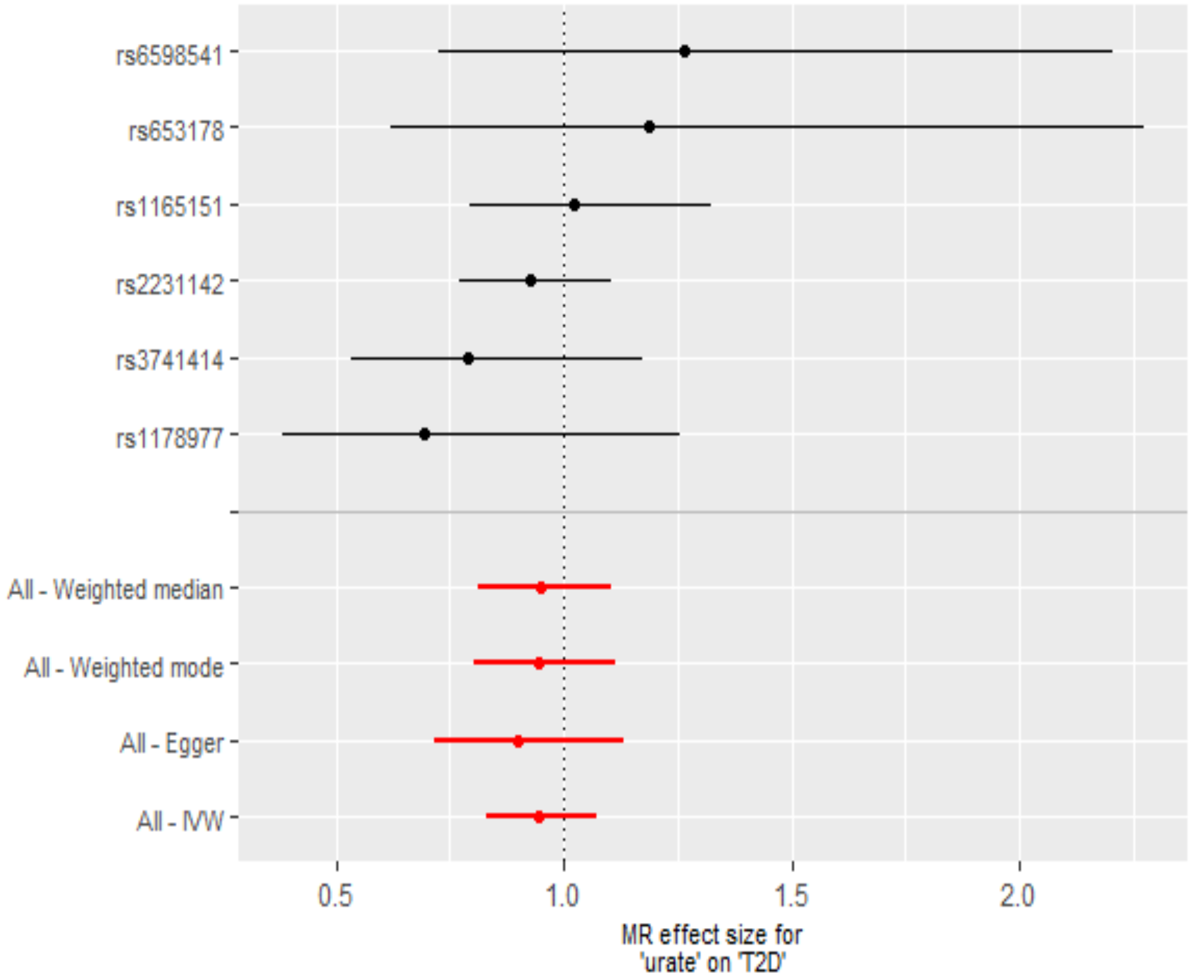

The y-axis contains the instrumental SNPs and meta-analyses of their Wald ratios: inverse-variance weighted (IVW; the main MR result) and sensitivity estimators (weighted median, weighted mode, and MR-Egger). The magnitude and direction of the effects of the sensitivity estimators are compared visually with those of the IVW. When the estimators align (as shown above), this is evidence against pleiotropy.

Supplementary figure 8. Scatter plot for MR of urate on type 2 diabetes (T2D).

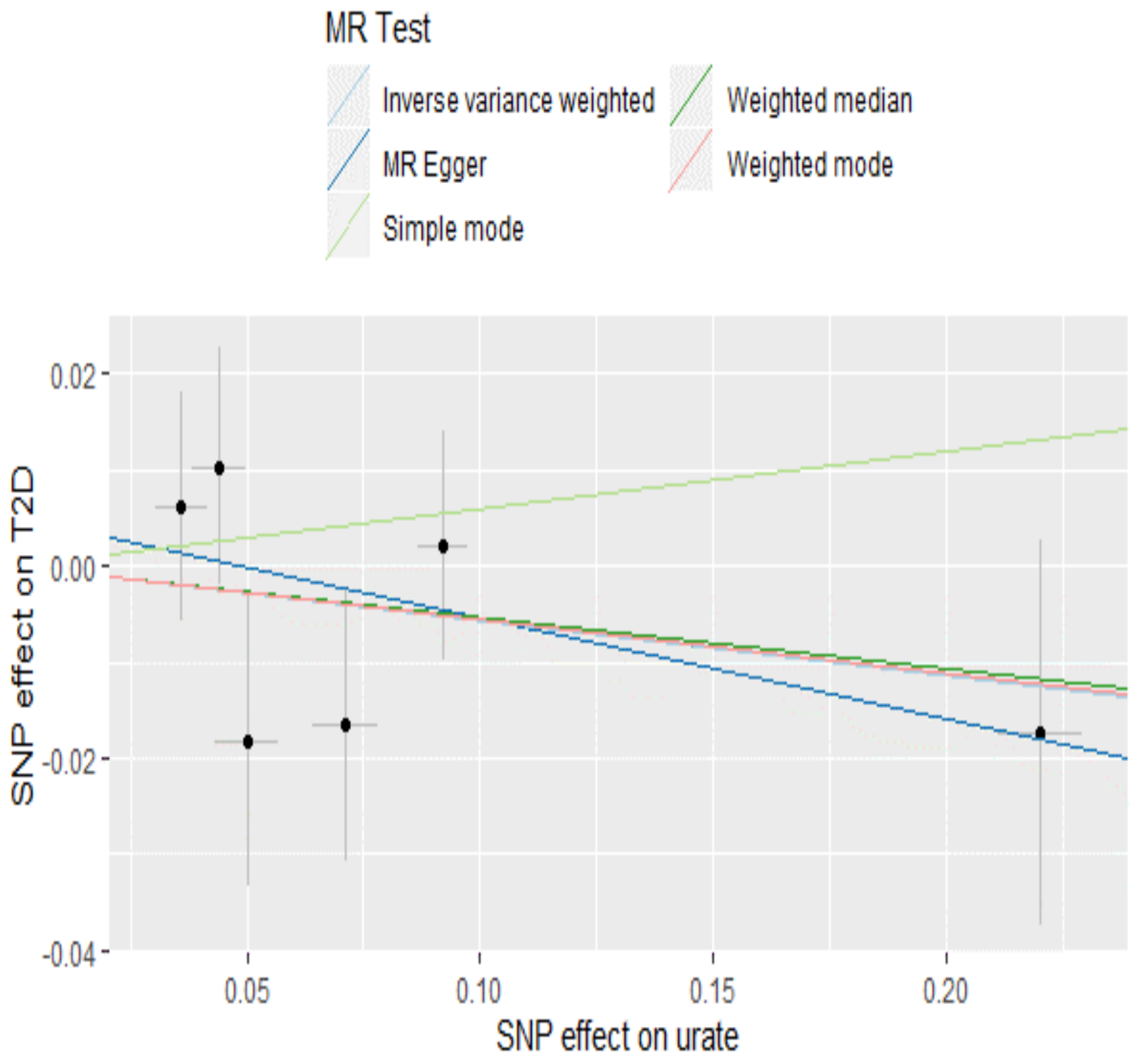

Supplementary figure 9. Forest plot for MR of obesity on high-density lipoprotein cholesterol (HDL).

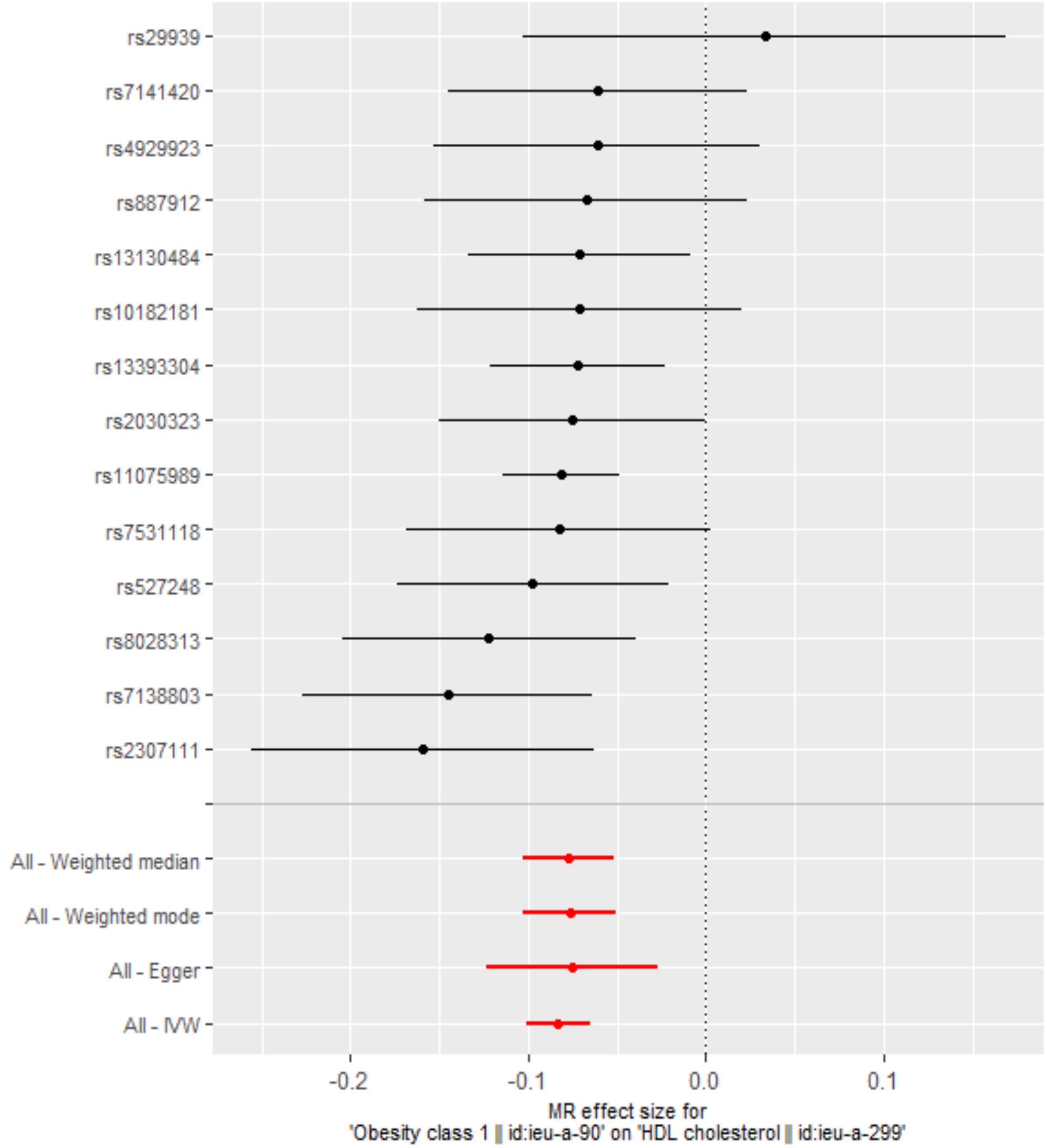

The y-axis contains the instrumental SNPs and meta-analyses of their Wald ratios: inverse-variance weighted (IVW; the main MR result) and sensitivity estimators (weighted median, weighted mode, and MR-Egger). The magnitude and direction of the effects of the sensitivity estimators are compared visually with those of the IVW. When the estimators align (as shown above), this is evidence against pleiotropy.

Supplementary figure 10. Scatter plot for MR of obesity on high-density lipoprotein cholesterol (HDL).

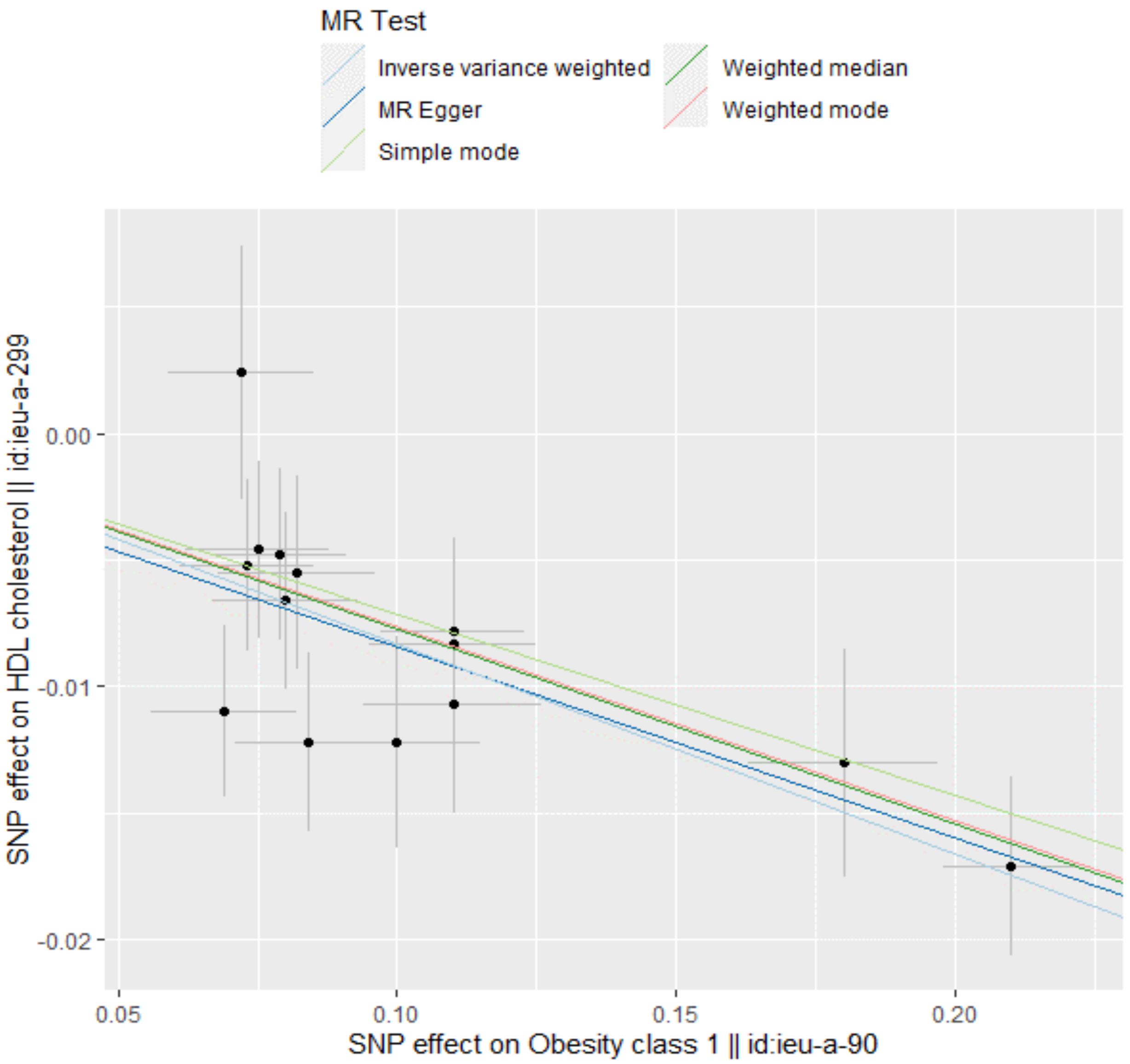

Supplementary figure 11. Forest plot for MR of obesity on low-density lipoprotein cholesterol (LDL).

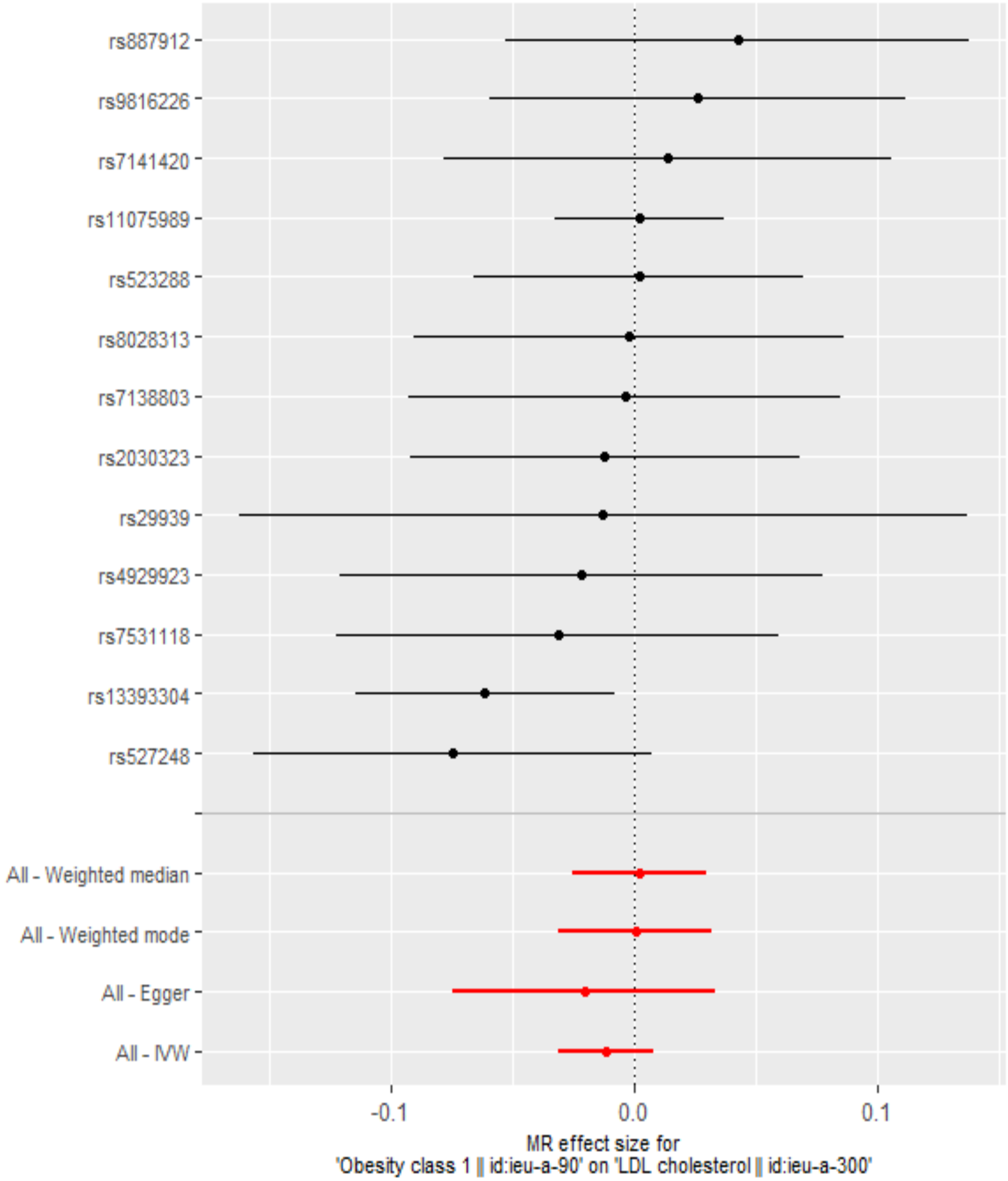

The y-axis contains the instrumental SNPs and meta-analyses of their Wald ratios: inverse-variance weighted (IVW; the main MR result) and sensitivity estimators (weighted median, weighted mode, and MR-Egger). The magnitude and direction of the effects of the sensitivity estimators are compared visually with those of the IVW. When the estimators align, this is evidence against pleiotropy. As shown above, the directions of the effects for the meta-analytic estimators are discrepant (some are <1 and some are 1).

Supplementary figure 12. Scatter plot for MR of obesity on low-density lipoprotein cholesterol (LDL).

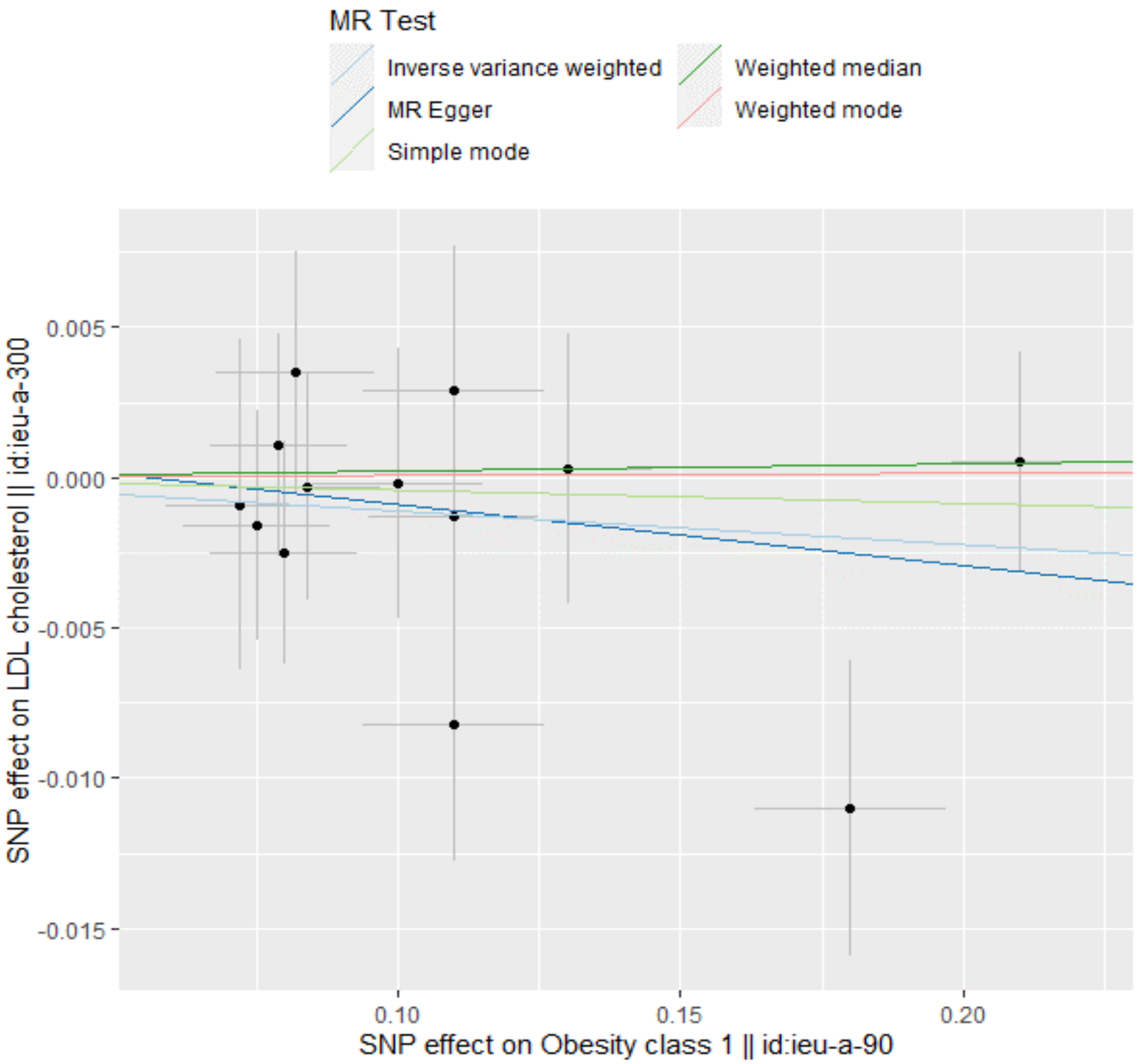

Supplementary figure 13. Forest plot for MR of obesity on triglycerides.

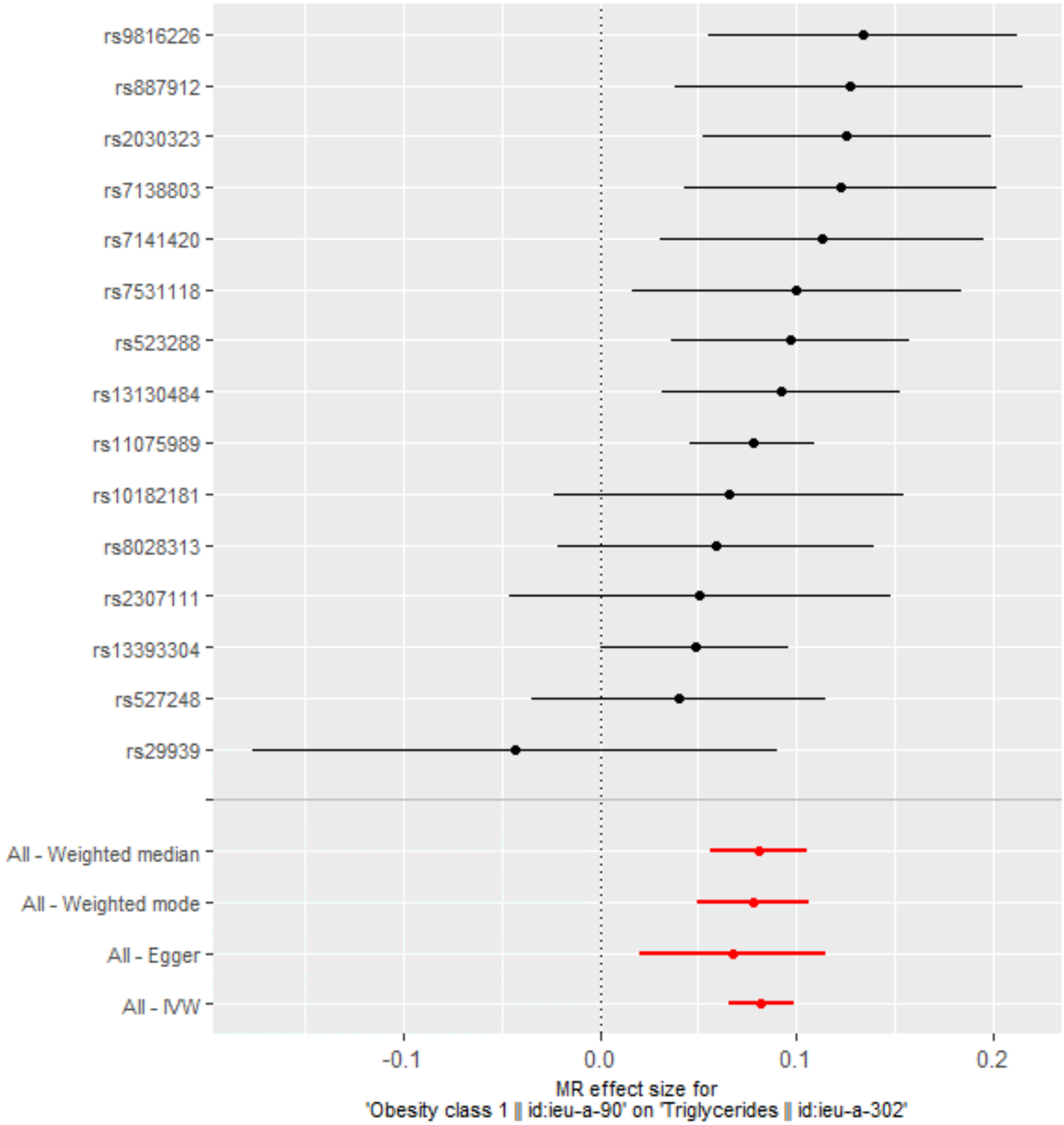

The y-axis contains the instrumental SNPs and meta-analyses of their Wald ratios: inverse-variance weighted (IVW; the main MR result) and sensitivity estimators (weighted median, weighted mode, and MR-Egger). The magnitude and direction of the effects of the sensitivity estimators are compared visually with those of the IVW. When the estimators align (as shown above), this is evidence against pleiotropy.

Supplementary figure 14. Scatter plot for MR of obesity on triglycerides.

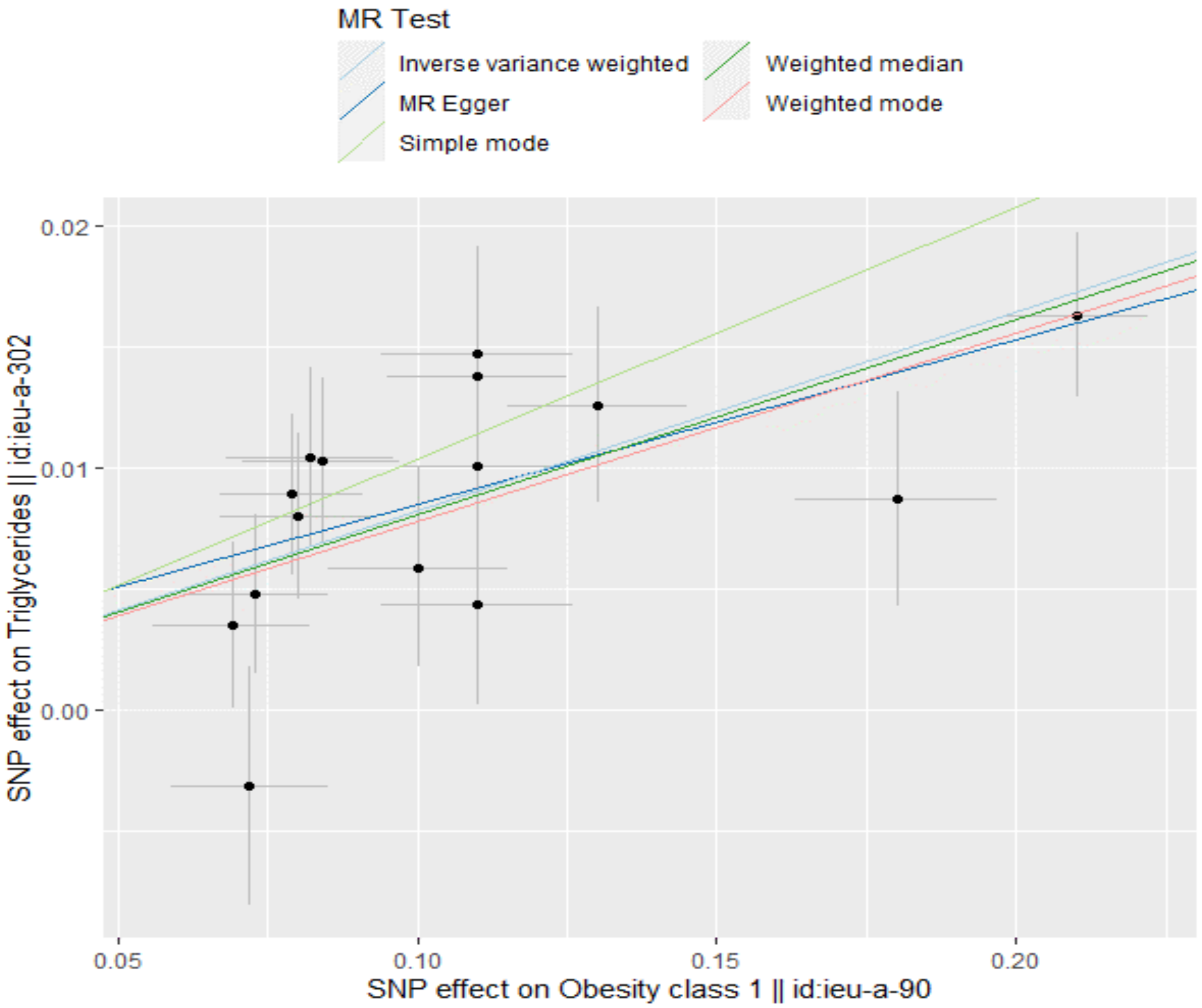

Supplementary figure 15. Forest plot for MR of high-density lipoprotein (HDL) on urate.

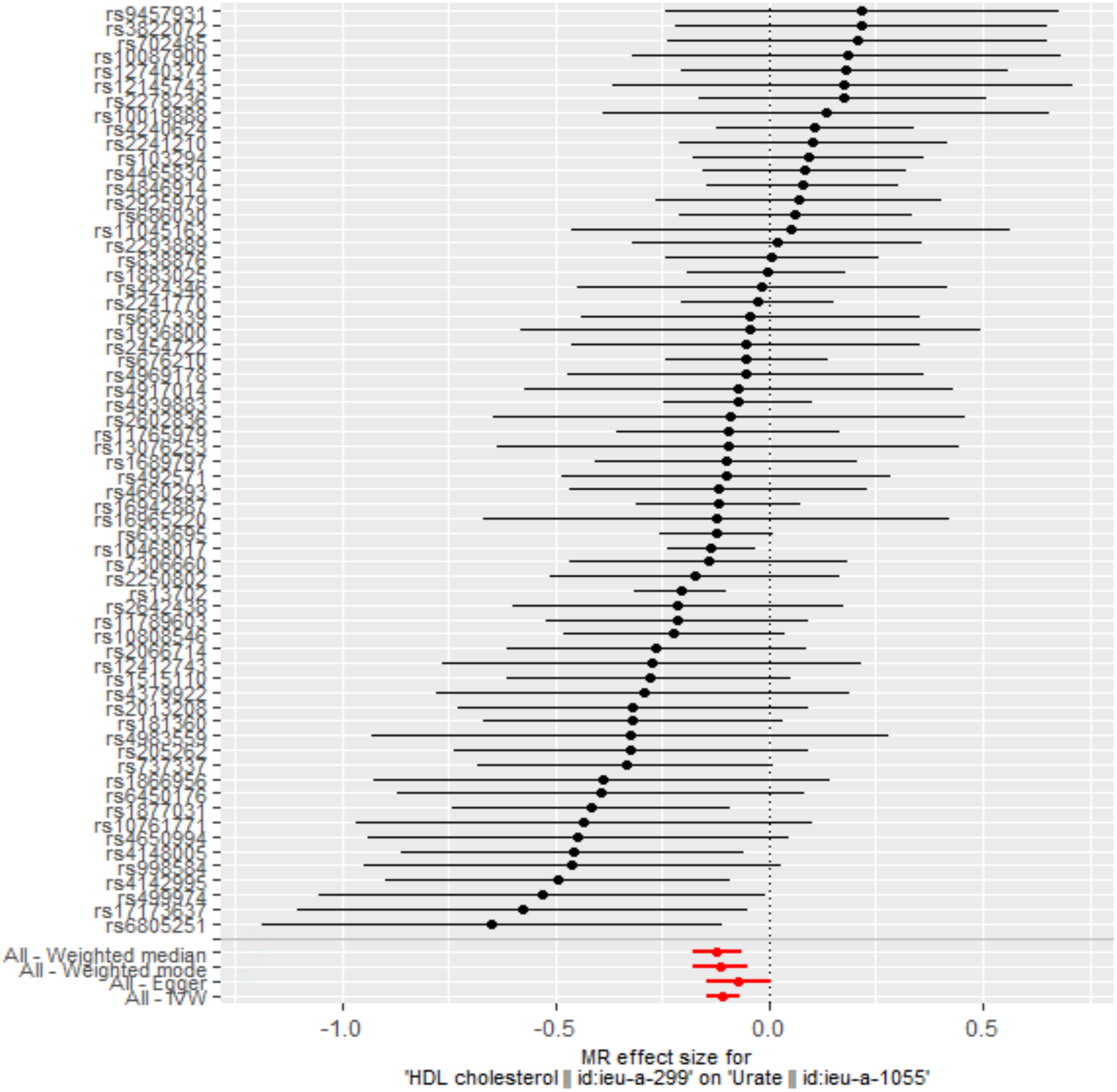

The y-axis contains the instrumental SNPs and meta-analyses of their Wald ratios: inverse-variance weighted (IVW; the main MR result) and sensitivity estimators (weighted median, weighted mode, and MR-Egger). The magnitude and direction of the effects of the sensitivity estimators are compared visually with those of the IVW. When the estimators align (as shown above), this is evidence against pleiotropy.

Supplementary figure 16. Scatter plot for MR of high-density lipoprotein (HDL) on urate.

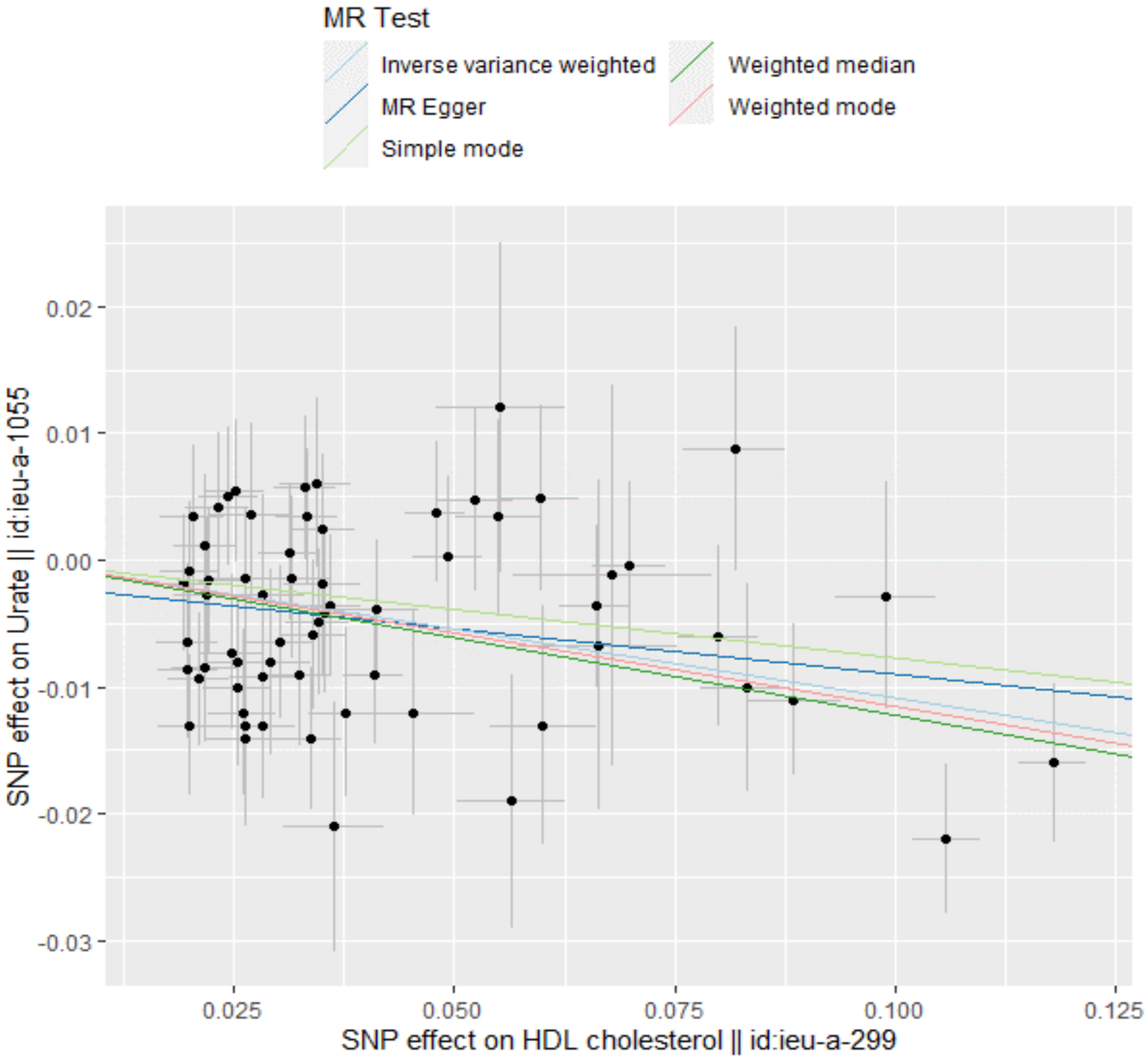

Supplementary figure 17. Forest plot for MR of triglycerides on urate.

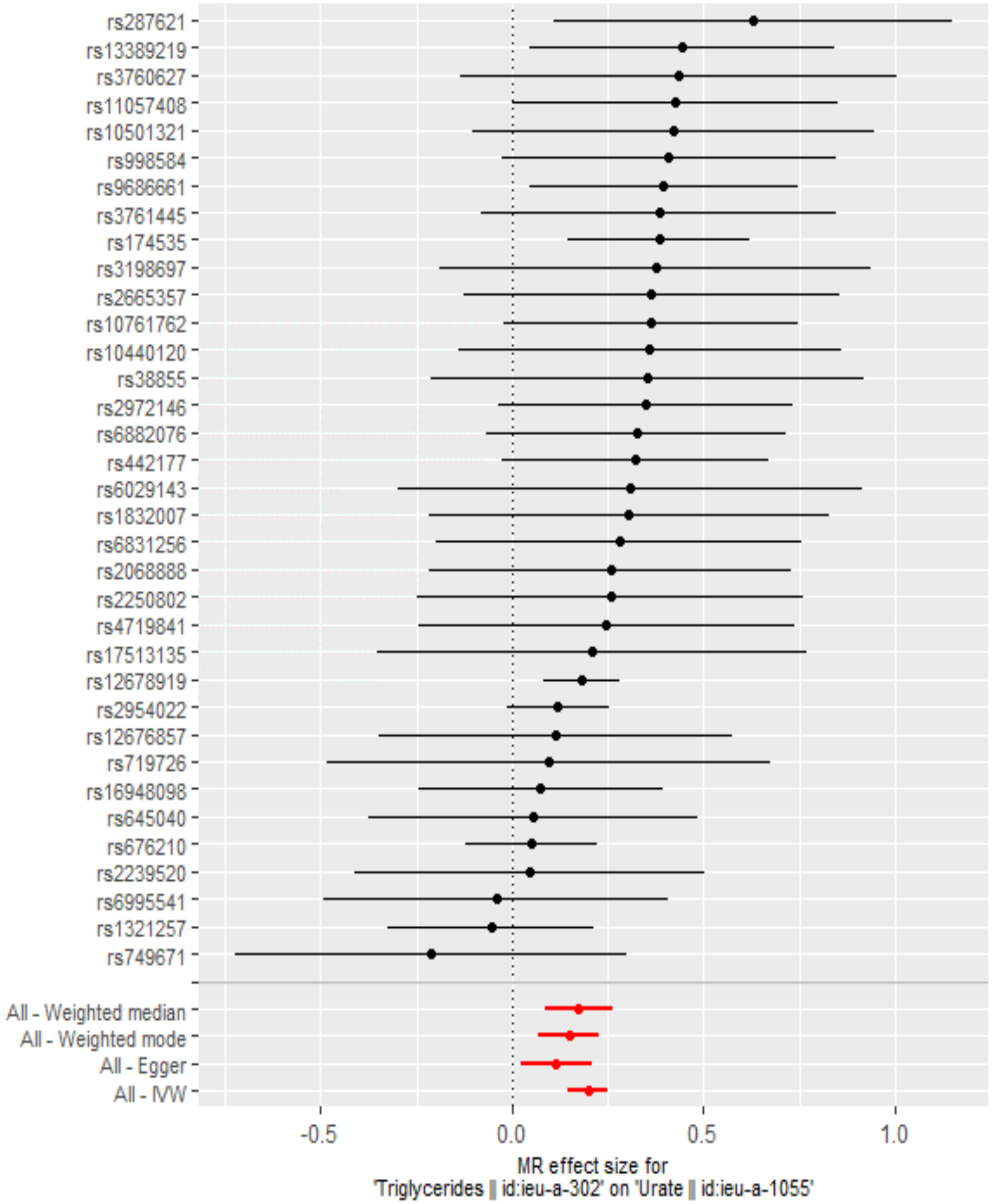

The y-axis contains the instrumental SNPs and meta-analyses of their Wald ratios: inverse-variance weighted (IVW; the main MR result) and sensitivity estimators (weighted median, weighted mode, and MR-Egger). The magnitude and direction of the effects of the sensitivity estimators are compared visually with those of the IVW. When the estimators align (as shown above), this is evidence against pleiotropy.

Supplementary figure 18. Scatter plot for MR of triglycerides on urate.

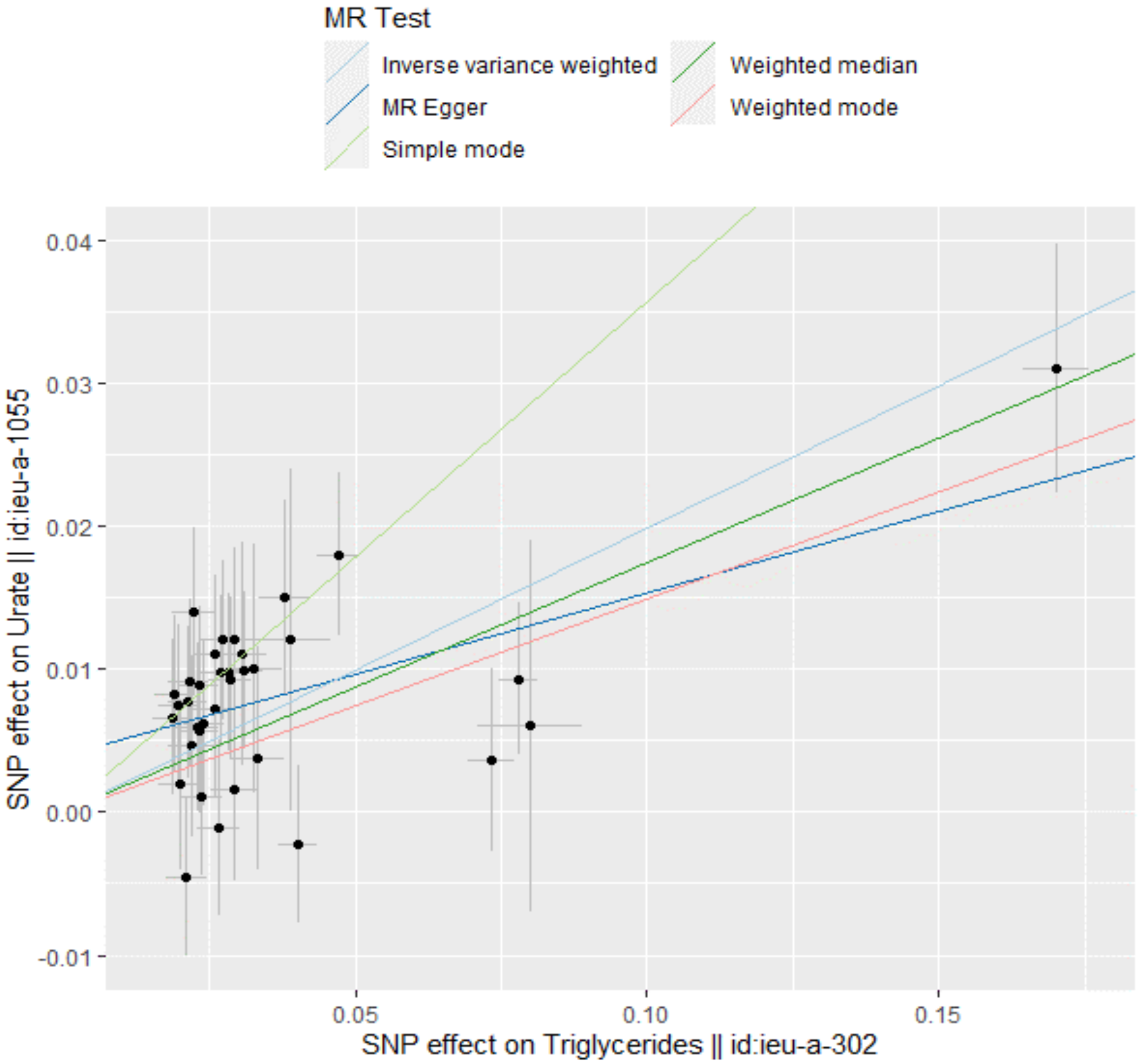

Supplement: Supplementary file 1 — Supplementary Figures. [file 41598_2021_97410_MOESM1_ESM.pdf]
